# Complex Mitochondrial Dysfunction Induced by TPP^+^-Gentisic Acid and Mitochondrial Translation Inhibition by Doxycycline Evokes Synergistic Lethality in Breast Cancer Cells

**DOI:** 10.3390/cells9020407

**Published:** 2020-02-11

**Authors:** Sebastián Fuentes-Retamal, Cristian Sandoval-Acuña, Liliana Peredo-Silva, Daniela Guzmán-Rivera, Mario Pavani, Natalia Torrealba, Jaroslav Truksa, Vicente Castro-Castillo, Mabel Catalán, Ulrike Kemmerling, Félix A. Urra, Jorge Ferreira

**Affiliations:** 1 Clinical and Molecular Pharmacology Program, Institute of Biomedical Sciences (ICBM), Faculty of Medicine, University of Chile, Santiago 8380453, Chile; sebastianfuentes@ug.uchile.cl (S.F.-R.);; 2 Institute of Biotechnology, Czech Academy of Sciences, 25250 Prague, Czech Republic; 3 School of Pharmacy, Faculty of Medicine, Andrés Bello National University, Santiago 8370149, Chile; 4 Department of Organic and Physical Chemistry, Faculty of Chemical and Pharmaceutical Sciences, University of Chile, Santiago 8380494, Chile; 5 Developmental Biology, Program of Anatomy, Institute of Biomedical Sciences (ICBM), Faculty of Medicine, University of Chile, Santiago 8380453, Chile

**Keywords:** inhibition of the electron transport chain, inhibition of alpha-ketoglutarate dehydrogenase complex, mitochondrially targeted, decyl polyhydroxybenzoate triphenylphosphonium derivatives, doxycycline, mitochondrial ribosome inhibition

## Abstract

The mitochondrion has emerged as a promising therapeutic target for novel cancer treatments because of its essential role in tumorigenesis and resistance to chemotherapy. Previously, we described a natural compound, 10-((2,5-dihydroxybenzoyl)oxy)decyl) triphenylphosphonium bromide (GA-TPP^+^C_10_), with a hydroquinone scaffold that selectively targets the mitochondria of breast cancer (BC) cells by binding to the triphenylphosphonium group as a chemical chaperone; however, the mechanism of action remains unclear. In this work, we showed that GA-TPP^+^C_10_ causes time-dependent complex inhibition of the mitochondrial bioenergetics of BC cells, characterized by (1) an initial phase of mitochondrial uptake with an uncoupling effect of oxidative phosphorylation, as previously reported, (2) inhibition of Complex I-dependent respiration, and (3) a late phase of mitochondrial accumulation with inhibition of α-ketoglutarate dehydrogenase complex (αKGDHC) activity. These events led to cell cycle arrest in the G1 phase and cell death at 24 and 48 h of exposure, and the cells were rescued by the addition of the cell-penetrating metabolic intermediates l-aspartic acid β-methyl ester (mAsp) and dimethyl α-ketoglutarate (dm-KG). In addition, this unexpected blocking of mitochondrial function triggered metabolic remodeling toward glycolysis, AMPK activation, increased expression of proliferator-activated receptor gamma coactivator 1-alpha (*pgc1α*) and electron transport chain (ETC) component-related genes encoded by mitochondrial DNA and downregulation of the uncoupling proteins *ucp3* and *ucp4*, suggesting an AMPK-dependent prosurvival adaptive response in cancer cells. Consistent with this finding, we showed that inhibition of mitochondrial translation with doxycycline, a broad-spectrum antibiotic that inhibits the 28 S subunit of the mitochondrial ribosome, in the presence of GA-TPP^+^C_10_ significantly reduces the mt-CO1 and VDAC protein levels and the FCCP-stimulated maximal electron flux and promotes selective and synergistic cytotoxic effects on BC cells at 24 h of treatment. Based on our results, we propose that this combined strategy based on blockage of the adaptive response induced by mitochondrial bioenergetic inhibition may have therapeutic relevance in BC.

## 1. Introduction

Recent research has shown that mitochondria and the correct assembly of the components of oxidative phosphorylation (OXPHOS) are required for tumor formation and metastasis [1,2]. In particular, cancer cells with high glycolytic metabolism exacerbate the expression of OXPHOS and mitochondrial biogenesis-related genes to supply ATP and superoxide to maintain the metastatic characteristics [1,3,4]. In this regard, tumor cells without mitochondrial DNA (mt-DNA) present a quiescent state and can only initiate the development of tumors and metastasis when they manage to reassemble their electron transport chain (ETC), either by incorporating mt-DNA or whole mitochondria from adjacent cells [5,6,7]. Consistent with the above findings, mitochondria have emerged as a promising target for anticancer strategies.

The capacity to support energy demand in cancer cells is promoted by mitochondrial biogenesis, where proliferator-activated receptor gamma coactivator 1-alpha (PGC1α) orchestrates the synthesis and assembly of different components of the ETC through the activation of transcription factors and mTOR/AMP-activated kinase (AMPK) prosurvival signaling [8]. This phenomenon determines the effect of several anticancer small molecules that have therapeutic targets related to OXPHOS [9,10]. Moreover, the metabolic heterogeneity of different subpopulations of cancer cells in the same tumor contributes to partial eradication/inhibition of tumor growth [11,12] and acquisition of resistance to chemotherapeutics [13,14]. Therefore, elucidation of the induction of the metabolism-dependent adaptive responses of cancer cells may be exploited to identify new targets to produce tumor vulnerability and overcome drug resistance to classical chemotherapeutics.

An extensively studied strategy for mitochondrial delivery of small molecules is the incorporation of lipophilic cationic groups such as pyridinium or triphenylphosphonium (TPP^+^), which allow the accumulation of lipophilic cationic-linked compounds specifically in the “negative” mitochondrial matrix based on the mitochondrial transmembrane potential (ΔΨm) [15,16,17]. Several TPP^+^-linked small molecules with anticancer effects, such as FDA-approved drugs, polyphenols, therapeutic peptides, and photosensitizers, have been reported [18]. We showed that TPP^+^-linked natural hydroxybenzoic acids induce OXPHOS inhibition and consistently decrease mitochondrial depolarization and ATP without increasing ROS production. Interestingly, these derivatives trigger mitochondrial metabolic stress, leading to the selective release of proapoptotic factors in several cancer cell lines, exhibiting antitumoral activities [19,20] with nonobservable toxicity in nontumoral tissues in vivo [21].

**Figure 1:**
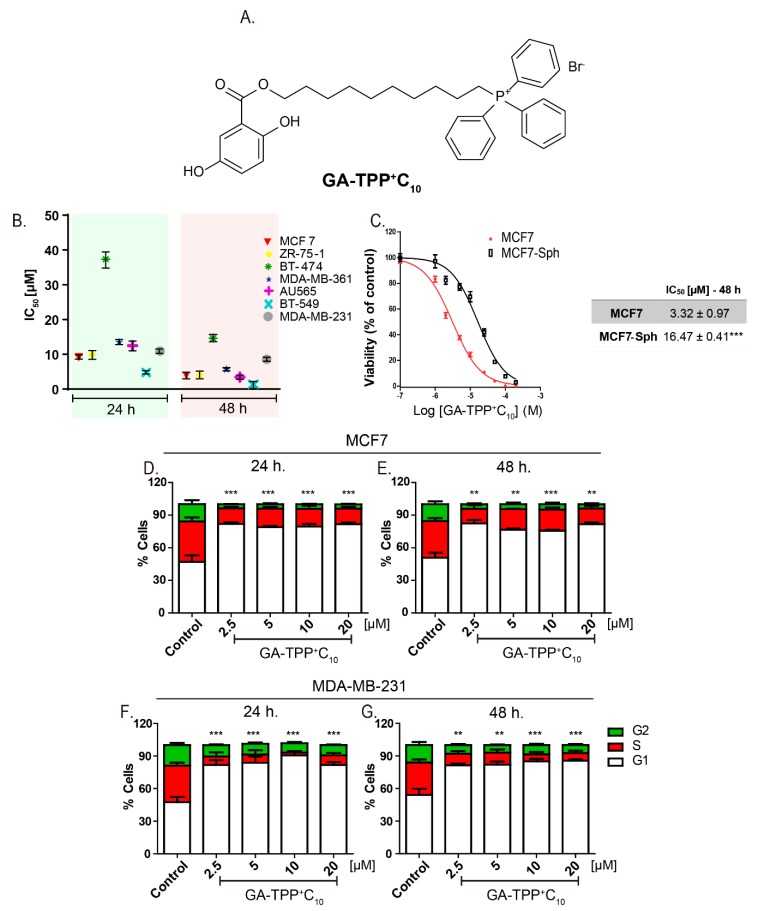
GA-TPP^+^C_10_ decreases the cell viability and produces cell cycle arrest in G_1_-phase breast cancer (BC) cells. (**A**) Chemical structure of 10-([(2,5-dihydroxybenzoyl)oxy]decyl) triphenylphosphonium bromide (GA-TPP^+^C_10_). (**B**) Effect of GA-TPP^+^C_10_ on the MTT reduction of a panel of BC cells after 24 and 48 h and in (**C**) MCF7 spheroids. (**D**,**E**) Effect of GA-TPP^+^C_10_ on cell cycle distribution after 24 and 48 h in MCF7 and (**F**,**G**) MDA-MB-231 cells, measured by flow cytometry. Values are expressed as the mean ± SEM of three independent experiments. ** *p* < 0.01, *** *p* < 0.001 vs. the G_1_-phase control.

Using 10-((2,5-dihydroxybenzoyl)oxy)decyl) triphenylphosphonium bromide (GA-TPP^+^C_10_, Figure 1A), we produced a time-dependent complex inhibition of the mitochondrial bioenergetics in breast cancer (BC) cell lines, triggering a prosurvival adaptive response dependent on the initial metabolic remodeling toward glycolysis and the enhanced expression of mitochondrial genes. Based on this result, we selected doxycycline (Doxy), a known antibiotic with a wide range of clinical applications [22,23], to inhibit protein synthesis by preventing the binding of activated tRNA to site A of the 28S subunit of mitochondrial ribosomes [24,25]. The exposure to GA-TPP^+^C_10_ plus Doxy led to selective and synergistic death via suppression of the cellular compensatory response in BC cells.

## 2. Materials and Methods

### 2.1. Compounds

The synthesis of GA-TPP^+^C_10_ was carried out according to Sandoval-Acuna et al. [20]. All stock solutions were prepared in dimethyl sulfoxide (DMSO) (Merck, Darmstadt, Germany).

### 2.2. Cell Lines and Cell Culture

The human BC cell lines MCF7 (ATCC HTB-22), ZR-75-1 (ATCC CRL-1599), BT-474 (ATCC HTB-20), BT-549 (ATCC HTB-122), MDA-MB-231 (ATCC CRM-HTB-26), AU565 (ATCC CRL-2351), and MDA-MB-361 (ATCC HTB-27) and the normal breast epithelial cell line MCF-10F (ATCC CRL-10318) were purchased from ATCC (ATCC, Manassas, VA, USA) and cultured in DMEM high-glucose medium [25 mM glucose and 4 mM glutamine, without pyruvate (Pyr), (Sigma Aldrich, St. Louis, MO, USA), promoting the same substrate availabilities for all cell lines. A description of the MCF7-TAMR, MCF7-rho0 and MCF7-Sph cells is provided in Appendix A.

### 2.3. MTT Reduction and Analysis of Isobolograms

The MTT assay was used to preliminarily evaluate the effect of GA-TPP^+^C_10_ (0.1–50 μM) and Doxy (1–1000 μM) on cellular proliferation using seven BC cell lines (MCF7, ZR-75-1, BT-474, BT-549, MDA-MB-231, AU565, MDA-MB-361) and nontumoral MCF-10F cells as previously reported by us [20], and the viability of the MCF7-Sph cells was evaluated by measuring the cellular ATP content using the CellTiter-Glo Luminescent Cell Viability Assay Kit (Promega, Madison, WI, USA) according to the manufacturer’s instructions. The construction and analysis of the isobolograms was carried out according to the values previously reported by Tallarida [26].

### 2.4. Crystal Violet Staining

MCF7 and MDA-MB-231 cell lines were incubated with different concentrations of GA-TPP^+^C_10_ for 24 h. Then, the culture medium was removed, and the cells were washed twice with PBS and incubated at room temperature for 30 min in 0.5% crystal violet and 20% methanol staining solution. Next, the plate was washed, inverted on filter paper to remove the remaining liquid and dried at room temperature for 3 h. The remnant crystal violet was solubilized with 200 μL of methanol per well. OD was measured at 570 nm using a Varioskan Flash^®^ microplate reader (Thermo Scientific, Waltham, MA, USA).

### 2.5. Colony Formation

For the colony assay, MCF7 and MDA-MB-231 cells were seeded in 6-well plates at 250 and 500 cells per well according to Franken, et al. [27] and incubated for 24 h. The cells were treated with GA-TPP^+^C_10_ for 24 h. After treatment, the medium was replaced with fresh medium, and the cells were incubated for 7 days to allow colony formation. Colonies were stained with crystal violet solution in 0.5% methanol and washed with tap water. Colony formation was analyzed with ImageJ software (NIH, Bethesda, MD, USA), and the surviving fraction was calculated according to Franken’s protocol [27].

### 2.6. Determination of Respiratory Complex-Dependent Respiration in Permeabilized Cancer Cells

In MCF7 BC cells (5 × 10^6^ cells), oxygen consumption was measured polarographically at 25 °C with a Clark electrode no. 5331 (Yellow Springs Instruments) using a YSI model 53 monitor connected to a 100-mV single-channel Goerz RE 511 recorder. The respiration buffer contained 200 mM sucrose, 50 mM KCl, 3 mM K_2_HPO_4_, 2 mM MgCl_2_, 0.5 mM EGTA, and 3 mM HEPES (pH 7.4). The MCF7 cells were incubated for 15 min with DMSO (control), GA-TPP^+^C_10_ (10 μM), gentisic acid (GA) (10 μM), or OH-C_10_TPP^+^ (10 μM), and the basal respiration rate was registered, followed by the addition of rotenone (3 μM), digitonin (10 μg/mL), and 5.0 mM succinate for Complex II at 6 min; antimycin A (3 μM), 1.5 mM ascorbate and 75 μM TMPD for Complex IV at 12 min; and finally 0.4 mM KCN at 24 min. For evaluation of Complex III-dependent respiration, permeabilized MCF7 cells in respiration buffer were treated with rotenone (3 μM), duroquinol (0.3 mM) at 12 min, and 0.4 mM KCN at 24 min as previously reported [28].

The inhibitory efficacy on Complex I-dependent respiration of each compound was analyzed by comparing the basal OCR versus rotenone-sensitive OCR.

### 2.7. Determination of ΔΨm and Mitochondrial and Intracellular ROS Levels

The effect of GA-TPP^+^C_10_ (10 μM) on ΔΨm was determined by using tetramethylrhodamine methyl ester (5 nM TMRM, in nonquenching mode, Molecular Probes, Molecular Probes, Eugene, OR, USA), and the mitochondrial and intracellular ROS levels were measured using the fluorescent probes MitoSOX (1 μM, Invitrogen, Waltham, MA, USA) and dihydroethidium (5 μM DHE, Sigma Aldrich), respectively, in MCF7 cells. Briefly, 1 × 10^5^ MCF7 cells were seeded on 12-well plates and incubated overnight. Next, GA-TPP^+^C_10_ (10 μM) was added to the cells, and the cells were incubated for 5, 15, 30, and 60 min. Then, the cells were incubated with the fluorescent probes for 15 min protected from light, and the changes in fluorescence were measured by flow cytometry as previously described [29].

### 2.8. Cellular Respiration and Extracellular Acidification Rate in Real Time

MCF7 and MDA-MB-231 BC cells (20,000 cells/well) were seeded on XFe96 V3-PS multiwell plates and kept overnight at 37 °C in 5% CO_2_ with culture medium containing glucose plus glutamine. For analysis of cellular respiration on the next day, the culture medium was replaced with assay medium (unbuffered DMEM without phenol red and with 4 mM glutamine and 10 mM glucose, pH 7.4) 1 h before the assay. Mitochondrial function was evaluated using 1 μM oligomycin, 50 nM FCCP, 1 μM rotenone, and 1 μM antimycin A. For analysis of glycolysis, the culture medium was replaced with assay medium (unbuffered DMEM without phenol red and with 4 mM glutamine, pH 7.4) 1 h before the assay. Glycolysis was evaluated by adding 10 mM glucose, 1 μM oligomycin, and 100 mM 2-deoxy-d-glucose (2-DG), as previously reported [29]. The oxygen consumption rate (OCR) and extracellular acidification rate (ECAR) measurements were made with the specific excitation and emission wavelengths of the fluorescent probes for oxygen (532/650 nm) and protons (470/530 nm). Each experiment was performed in triplicate.

### 2.9. Annexin V/Propidium Iodide Staining and Cell Cycle Analysis

The cell death induced in MCF7, MDA-MB-231 and MCF-10F cells by GA-TPP^+^C_10_ (2.5 μM), Doxy (10, 25, and 50 μM) and the GA-TPP^+^C_10_ plus Doxy combination was evaluated using annexin V/propidium iodide (AV/PI) dual staining, following the instructions of the Annexin V-FITC Apoptosis Detection Kit (Abcam, Cambridge, UK), as previously reported [20]. For evaluation of the effect of mitochondrial bioenergetic inhibition induced by GA-TPP^+^C_10_ on the viability of BC cells, MCF7 and MDA-MB-231 cells were treated with Pyr (5 mM) and the cell-penetrating metabolic intermediates l-aspartic acid β-methyl ester (mAsp, 5 mM) and dimethyl α-ketoglutarate (dm-KG, 5 mM) for 24 h; then, they were exposed to GA-TPP^+^C_10_ (10 μM) for 48 h, and cell death was determined.

For estimation of the cell cycle distribution, cellular DNA levels were measured by flow cytometry as previously described [28]. The BC cells were incubated with DMSO (control) or 2.5, 5, 10, and 20 μM GA-TPP^+^C_10_ for 24 and 48 h. All samples were analyzed for cell cycle distribution using a FACSCalibur flow cytometer and Becton–Dickinson CellQuest acquisition software (San Jose, CA, USA).

### 2.10. α-Ketoglutarate Dehydrogenase (αKGDH) Complex Activity Assay

The αKGDH complex activity was measured using the KGDH Activity Assay Colorimetric Kit (K678, BioVision, Milpitas, CA, USA). Briefly, 1 × 10^5^ MCF7 and MDA-MB-231 cells were seeded on 6-well plates and incubated overnight. Next, GA-TPP^+^C_10_ (5 and 10 μM) was added to the cancer cells, and the cells were incubated for 24 h. Subsequently, the cells were trypsinized and lysed. Subsequent treatment of the samples and measurements were performed according to the manufacturer’s instructions. The absorbance value was measured using a Varioskan Flash^®^ microplate reader (Thermo Scientific, Waltham, MA, USA). Michaelis-Menten fitting and kinetics constants were obtained using GraphPad Prism software (version 5.03, San Diego, CA, USA).

### 2.11. RNA Extraction, Reverse Transcription and qPCR

Evaluation of the effect of GA-TPP^+^C_10_ (5 μM) on the levels of different mRNAs was performed by qPCR, as described by Truksa et al. [30]. Total RNA was extracted using the RNAzol T Kit (Molecular Research Center, Inc., Cincinnati, OH, USA) according to the manufacturer’s instructions. qPCR analysis was performed using the Illumina Eco Real-Time PCR System (Illumina, San Diego, CA, USA). A detailed methodology and the primers used are provided in Appendix A.

### 2.12. Western Blotting

The total levels and phosphorylation at Threo172 of AMPKα in BC cells and nonmalignant cells treated with GA-TPP^+^C_10_ for 4 and 24 h and the levels of mitochondrial proteins (mt-ND_1_, mt-CO_1_, mt-CO_2_, UQCRC2, and VDAC1) induced by GA-TPP^+^C_10_ and Doxy at 24 h of exposure were also analyzed by Western blotting. The detailed methodology for blotting is provided in Appendix A.

### 2.13. Statistics

The results are expressed as the mean ± SEM of at least three independent experiments. The comparison between the different experimental groups and their respective controls was performed by one-way ANOVA (followed by Bonferroni post hoc analysis) using GraphPad Prism 5.0 software. *p* < 0.05 was established as the minimum significance level.

## 3. Results

### 3.1. GA-TPP^+^C_10_ Decreases the Clonogenic Potential and Viability, Producing Cell Cycle Arrest in G_1_-Phase BC Cells

The compound GA-TPP^+^C_10_ reduced cell proliferation in a time- and dose-dependent manner in the seven BC cell lines with IC_50_ values close to 10 μM (Figure 1B). Notably, BT-474 cells (ER+/HER2/neu+, p53 E285K) showed high resistance to the effect of GA-TPP^+^C_10_ because their IC_50_ values at 24 and 48 h were significantly higher than those observed in the other cell lines (Figure 1B). BC cells grown as spheres (MCF7-Sph) showed lower sensitivity to the antiproliferative effects than the parental MCF7 cells after 48 h of treatment with GA-TPP^+^C_10_ (Figure 1C). Consistent with the above finding, this compound produced significant cell cycle arrest in the G1 phase at 24 and 48 h (Figure 1D–G) and reduced the clonogenic potential in the MCF7 and MDA-MB-231 cells (Figure S1). Using four chemotherapeutics with different mechanisms of action, we treated the MCF7 and MDA-MB-231 cells with GA-TPP^+^C_10_ and alternatively with doxorubicin and etoposide (topoisomerase II inhibitors), imatinib (c-kit and/or PDGFR inhibitor [31]), and bleomycin (a DNA damage inductor). Then, the effect on viability was evaluated after 24 h of exposure. Notably, GA-TPP^+^C_10_ promoted vulnerability to cell death in the BC cells treated with all chemotherapeutics (Figure S2), suggesting that the mechanism of action of this compound blocks an essential metabolic adaptive response.

**Figure 2:**
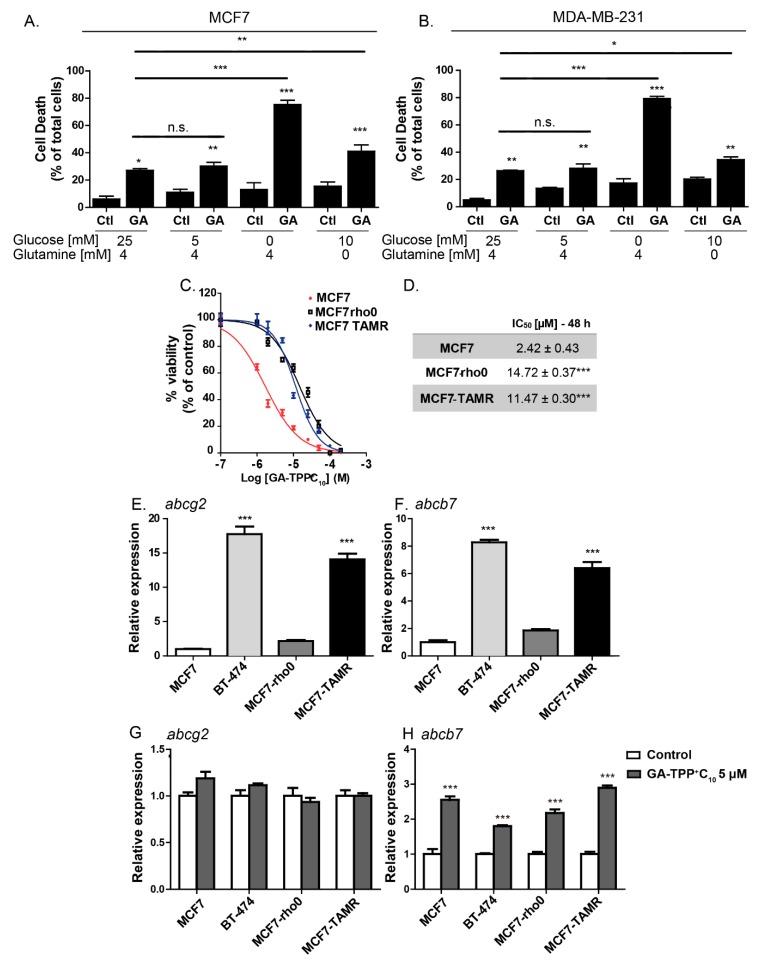
The anticancer effect of GA-TPP^+^C_10_ relies on substrate availability and promotes increased expression of the mito-ATP-binding cassette (ABC) transporter. (**A**) Effect of 10 μM GA-TPP^+^C_10_ on cell death induction in the MCF7 and (**B**) MDA-MB-231 cells cultured in different conditions of glucose and glutamine availability. The BC cells were grown in conditioned culture medium with different substrate availabilities 24 h before GA-TPP^+^C_10_ treatment. (**C**,**D**) Decreased viability induced by GA-TPP^+^C_10_ in wild-type MCF7 cells, cells devoid of mitochondrial DNA (MCF7-rho0) and cells with reduced mitochondrial bioenergetics (tamoxifen-resistant MCF7-TAMR). (**E**–**H**) Basal mRNA expression and changes induced by GA-TPP^+^C_10_ after 24 h in ATP-dependent transporters in the MCF7, BT-474, MCF7-rho0, and MCF7-TAMR cells, as measured by qPCR. Values are expressed as the mean ± SEM of three independent experiments. * *p* < 0.05, ** *p* < 0.01, *** *p* < 0.001 vs. the control or MCF7.

To evaluate whether the effect of GA-TPP^+^C_10_ is determined by the bioenergetic profile of BC cells, we modified the glucose and glutamine availabilities, and cell death was evaluated. As shown in Figure 2A,B, a similar cytotoxic effect was observed in the presence of 5 and 25 mM glucose, but a discrete and significant increase in cell death was produced by glutamine deprivation. By completely substituting glucose for galactose, we generated BC cell subpopulations that exhibited high dependence on respiration and decreased involvement of glycolysis to meet the energy demand [29]. Under this condition, GA-TPP^+^C_10_ produced extensive cell death. Additionally, MCF7-rho0 cells, which lack mitochondrial DNA and consistently exhibit reduced mitochondrial gene expression (Figure S3), exhibited a 4-fold increase in resistance to GA-TPP^+^C_10_, similar to tamoxifen-resistant BC cells (MCF7-TAMR) (Figure 2C,D), a cell line with a decrease in mitochondrial respiration and a low abundance of the assembled mitochondrial respiratory supercomplexes [32]. Altogether, our results suggest that GA-TPP^+^C_10_ has as a primary target the mitochondrial ETC in BC cells.

### 3.2. The Effect of GA-TPP^+^C_10_ Increases the Gene Expression of Mitochondrial ABC Transporters

One of the mechanisms of resistance to cytotoxic compounds is overexpression of ATP-binding cassette (ABC) transporters [33,34]. Given that the cytosolic transporter ABCG2 has substrate compounds formed by alkyl triphenylphosphonium groups [33] and that the mitochondrial transporter ABCB7 plays an essential role in the function of the ETC Complexes I, II, III, and IV [35], we studied the changes in gene expression of these ABC transporters induced by GA-TPP^+^C_10_. Therefore, we studied the changes in gene expression of the cytosolic and mitochondrial ABC transporters triggered by the effect of GA-TPP^+^C_10_. First, we analyzed the basal expression of the cytosolic transporter *abcg2* (also known as breast cancer resistance protein 1; BCRP1) and the mitochondrial protein *abcb7* [33,36]. Figure 2E,F shows that the expression of *abcg2* and *abcb7* in BT-474 cells was 17 and 8 times higher, respectively, than that observed in the MCF7 cells. Additionally, when comparing these two transporters in the parental line MCF7 (ER+) versus its derivative resistant to tamoxifen (MCF7-TAMR), we found that the basal expression of *abcg2* and *abcb7* increased 14 and 6.5 times, respectively. In turn, GA-TPP^+^C_10_ significantly increased the expression of *abcb7* without altering the expression of *abcg2* in both the MCF7 and BT-474 cells. A similar effect was observed in the modified MCF7-rho0 and MCF7-TAMR cells (Figure 2G,H), suggesting that elevated gene expression of mitochondrial ABC transporters may be associated with mitochondrial uptake of GA-TPP^+^C_10_ in all cancer cells.

### 3.3. Acute GA-TPP^+^C_10_ Treatment Induces a Complex Inhibition of Mitochondrial Functions, Leading to Remodeling toward Glycolysis

**Figure 3:**
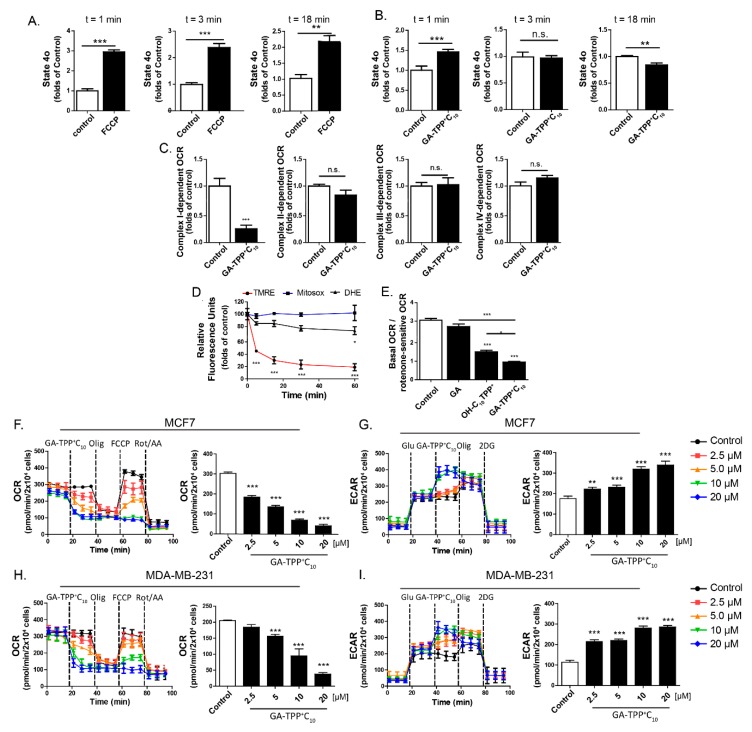
GA-TPP^+^C_10_ inhibits Complex I-dependent respiration and leads to a glycolytic phenotype in BC cells. (**A**) Effect of 1 μM FCCP and (**B**) 10 μM GA-TPP^+^C_10_ on the oxygen consumption rate (OCR) after 1–18 min in the apparent 4o state of the intact MCF7 cells. (**C**) Effect of 10 μM GA-TPP^+^C_10_ on each respiratory complex-dependent OCR in the permeabilized MCF7 cells using a Clark electrode. (**D**) Effect of 10 μM GA-TPP^+^C_10_ on ΔΨ_m_ and mitochondrial and intracellular ROS production in MCF7 cells using TMEM and DHE probes, respectively. (**E**) Effect of 10 μM GA-TPP^+^C_10_, gentisic acid, and OH-C_10_TPP^+^ on Complex I-dependent OCR. (**F**,**G**) GA-TPP^+^C_10_ decreases the maximal OCR and increases the glycolysis-dependent extracellular acidification rate (ECAR) value in MCF7 and (H-I) MDA-MB-231 cells, measured with a Seahorse system. Values are expressed as the mean ± SEM of three independent experiments. * *p* < 0.05, ** *p* < 0.01, *** *p* < 0.001 vs. the control (DMSO).

Previously, we showed that TPP^+^C_10_-linked polyphenols instantly produce an increase in mitochondrial respiration in state 4o [19], suggesting a possible uncoupling effect of OXPHOS in cancer cells [20,21]; however, the mechanism and site of action of these compounds in mitochondria remain uncertain. Therefore, we evaluated the effect of GA-TPP^+^C_10_ on oligomycin-insensitive respiration (apparent state 4o) in the MCF7 and MDA-MB-231 BC cells three times after exposure. As shown in Figure 3A,B, the protonophore FCCP produced a sustained increase in state 4o during 18 min of measurement. In contrast, GA-TPP^+^C_10_ produced a biphasic effect on respiration in state 4o. First, GA-TPP^+^C_10_ increased respiration (time = 0–1 min) and then produced a significant decrease in respiration in both BC cell lines. These effects were accompanied by a sustained Δψm drop without mitochondrial and intracellular ROS production, with the latter measured using MitoSOX and DHE assays, respectively (Figure 3D and Figure S4). To determine whether the decrease in respiration was produced by a direct interaction between GA-TPP^+^C_10_ and ETC, we evaluated respiratory complex-dependent respiration in the permeabilized MCF7 cells. At 15 min of exposure, GA-TPP^+^C_10_ inhibited only Complex I-dependent respiration (Figure 3C). Given that alkyltriphenylphosphonium cations decrease mitochondrial OCR through ETC inhibition [37,38,39] and that *p*-hydroquinones, similar to GA, inhibit Complex I-dependent respiration [10,28], we compared the inhibitory efficacy on Complex I-dependent respiration by using GA-TPP^+^C_10_ with GA and (10-hydroxydecyl) triphenylphosphonium bromide (OH-C_10_TPP^+^), both chemical fragments that constitute the mitochondria-targeted studied compound. As shown in Figure 3E, OH-C_10_TPP^+^ and GA had much lower inhibitory efficacy against rotenone-sensitive respiration than GA-TPP^+^C_10_, suggesting that this compound exhibits improved activity on the mitochondrial bioenergetics of cancer cells.

Consistent with this finding, GA-TPP^+^C_10_ in intact BC cells inhibited basal and maximal mitochondrial respiration and increased glycolysis-dependent ECAR values in a concentration-dependent manner (Figure 3F–I, Figure S5). Notably, blocking the glycolytic pathway with the hexokinase inhibitor 2-deoxyglucose (2-DG) produced a significant increase in the cell death induced by GA-TPP^+^C_10_, indicating that BC cell survival under GA-TPP^+^C_10_-induced respiration inhibition is energetically compensated by glycolysis (Figure S6).

### 3.4. Prolonged GA-TPP^+^C_10_ Treatment Induces Mixed Inhibition of the α-Ketoglutarate Dehydrogenase Complex (αKGDHC)

**Figure 4:**
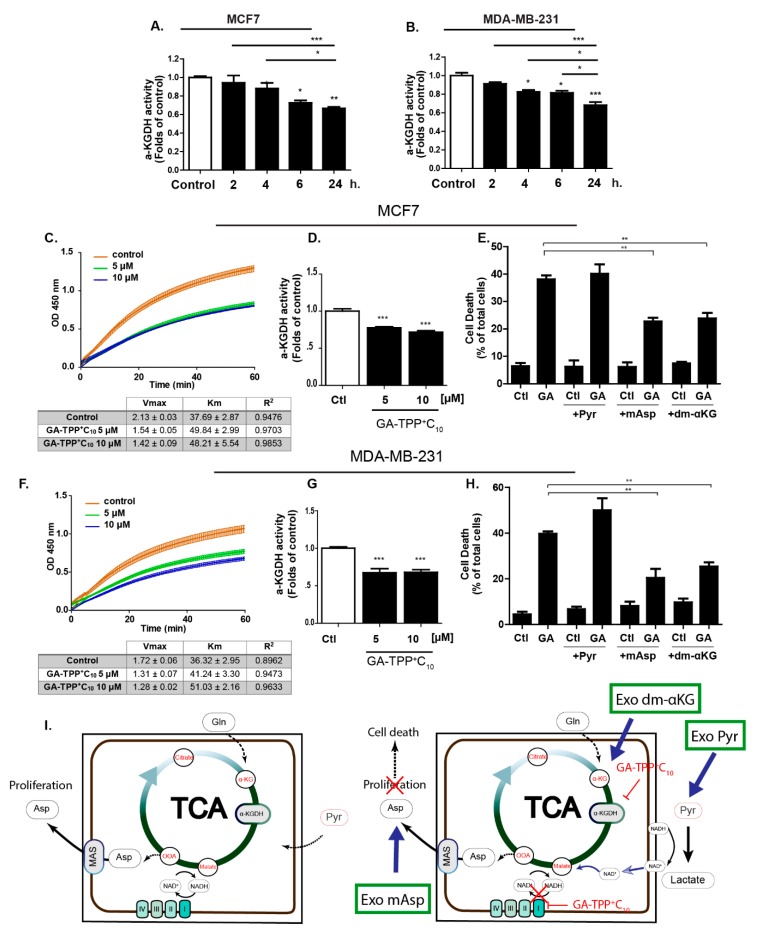
GA-TPP^+^C_10_ decreases alpha-ketoglutarate dehydrogenase (α-KGDH) activity in BC cells. (**A**,**B**) Effect of GA-TPP^+^C_10_ (10 μM) on the α-KGDH activity in BC cells at different times of exposure, (**C**,**D**) Effect of GA-TPP^+^C_10_ (10 μM) on the kinetic parameters of α-KGDH at 24 h of exposure in the MCF7 and (**F**,**G**) MDA-MB-231 cells; Vmax and km were obtained using Michaelis-Menten fitting. (**E**,**H**) Rescue of the cytotoxic effect of GA-TPP^+^C_10_ (GA, 10 μM) by L-aspartic acid β-methyl ester (mAsp, 5 mM) and dimethyl α-ketoglutarate (dm-KG, 5 mM) but not pyruvate (Pyr, 5 mM) in BC cells after 48 h. Viability was measured by flow cytometry. (**G**) Rescue scheme for the exogenous addition of the cell-penetrating metabolic intermediates pyruvate (Exo pyr), aspartate (Exo mAsp) and αKG (Exo dm-αKG). Values are expressed as the mean ± SEM of three independent experiments. * *p* < 0.05, ** *p* < 0.01, *** *p* < 0.001 vs. the control (DMSO).

Interestingly, the accumulation of alkyl-TPP^+^ cations in the mitochondrial matrix was shown to promote the inhibition of the activity of the αKGDHC [37], a tricarboxylic acid (TCA) cycle enzyme essential for glutaminolysis. Therefore, we evaluated whether GA-TPP^+^C_10_ treatment inhibits αKGDHC activity in BC cells at different times of exposure. As shown in Figure 4A,B, this compound decreases αKGDHC activity from 6 to 24 h of incubation, an event that occurs after Complex I-dependent OCR inhibition. Prolonged GA-TPP^+^C_10_ treatment (24 h) modified the kinetic parameters of αKGDHC, decreasing the Vmax and increasing the Km at 5 and 10 μM, indicating a mixed-inhibition model, as suggested by the Michaelis-Menten modeling (Figure 4C,D,F,G). The coordinated function of Complex I and αKGDHC is required for maintaining the mitochondrial NAD^+^/NADH ratio, which is essential for the synthesis of aspartate [40,41,42]. To determine whether the inhibition of Complex I and αKGDHC by GA-TPP^+^C_10_ has implications in the anticancer effect exhibited in BC cells, we added the exogenous metabolic substrate Pyr and the cell-penetrating intermediates mAsp and dm-KG, and the viability in the presence of GA-TPP^+^C_10_ was evaluated at 48 h of exposure. Pyr addition was shown to rescue the antiproliferative effect only of ETC inhibitors [40,41,42] by lactate dehydrogenase-dependent NADH regeneration, and mAsp and dm-αKG addition rescued the carbon source for nucleotide biosynthesis and αKGDHC activity-dependent glutaminolysis [29,43,44]. As shown in Figure 4D,G, consistent with the αKGDHC activity assay, both dm-αKG and mAsp, but not Pyr, partially rescued the cytotoxic effect of GA-TPP^+^C_10_ in the MCF7 and MDA-MB-231 cancer cells, suggesting that inhibition of αKGDHC activity is a relevant step for the promotion of mitochondrial dysfunction. Taken together, our results describe GA-TPP^+^C_10_ as a compound with three phases of interaction with mitochondria in a time-dependent manner: (1) initial phase of mitochondrial uptake with an uncoupling effect, as previously reported [20], (2) inhibition of Complex I-dependent respiration, and (3) late phase of mitochondrial accumulation with inhibition of αKGDHC activity (Figure 4I).

### 3.5. GA-TPP^+^C_10_ Induces Prosurvival AMPK Activation in BC Cells

**Figure 5:**
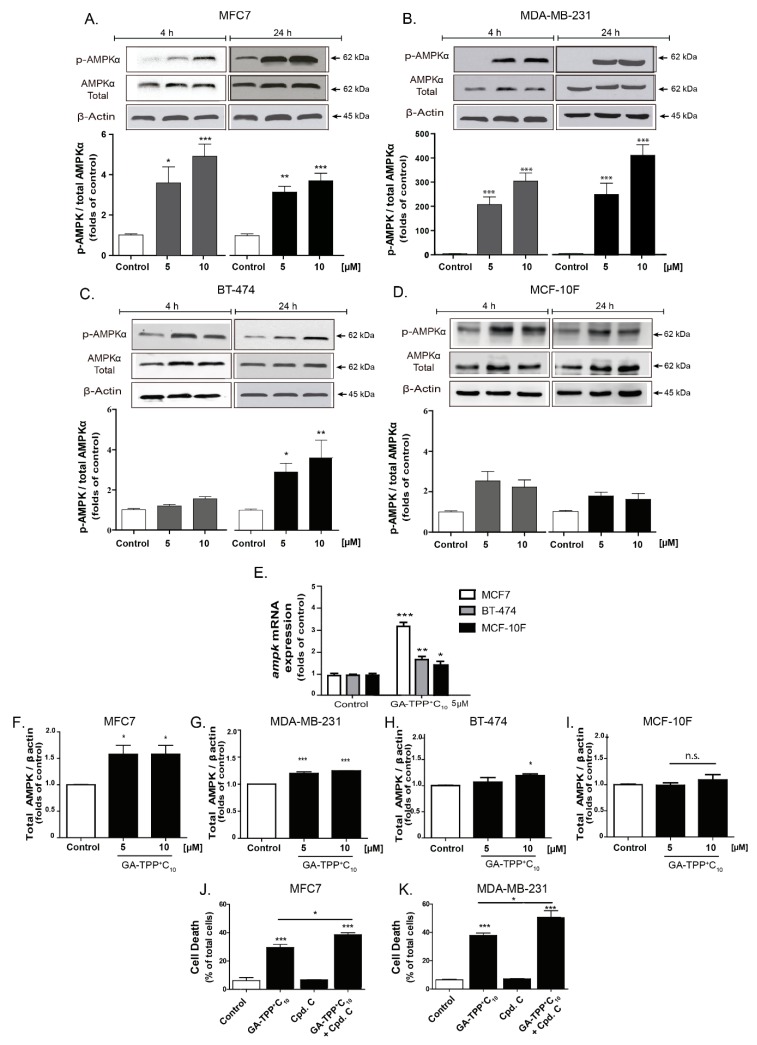
Prosurvival AMPK activation induced by GA-TPP^+^C_10_. (**A**–**D**) Phospho-AMPK levels induced by GA-TPP^+^C_10_ at 4 and 24 h of exposure in the BC cells and the epithelial cells. (**E**–**I**) Increased *ampk* mRNA and protein expression induced by GA-TPP^+^C_10_ in the BC cells and the breast epithelial cells after 24 h of exposure. (**J**,**K**) Effect of the decreased prosurvival AMPK on viability in the MCF7 and MDA-MB-231 cells incubated with GA-TPP^+^C_10_ after 24 h. Values are expressed as the mean ± SEM of three independent experiments. * *p* < 0.05, ** *p* < 0.01, *** *p* < 0.001 vs. the control (DMSO).

OXPHOS inhibition can trigger metabolic stress signaling mediated by energetic sensors such as AMPK [45,46]. As shown in Figure 5A,B, GA-TPP^+^C_10_ activates AMPK in the MCF7 and MDA-MB-231 cells, as evidenced by the quantification of its active form (phospho-AMPK) at 4 and 24 h of exposure.

Only after 24 h of incubation did we observe a significant increase in the active form of AMPK in the BT-474 cell line, which was highly resistant to GA-TPP^+^C_10_ (Figure 5C). No AMPK activation was induced by GA-TPP^+^C_10_ in the MFC-10F cells (Figure 5D). Although GA-TPP^+^C_10_ induced a significant increase in the mRNA levels of *ampk* in the BC and nontumoral cells, with a major effect in the MCF7 cells (Figure 5E), increased protein levels of AMPK were detected only in the BC cells (Figure 5F–I). In addition, the AMPK inhibitor compound C (Cpd. C, 2.5 μM) did not affect the viability of the BC cells; however, the combination of Cpd. C plus GA-TPP^+^C_10_ (10 μM) increased cell death (Figure 5J,K), suggesting that GA-TPP^+^C_10_ activates prosurvival AMPK signaling.

### 3.6. The Effect of GA-TPP^+^C_10_ Increases Mitochondrial Biogenesis-Related Gene Expression in BC Cells

**Figure 6:**
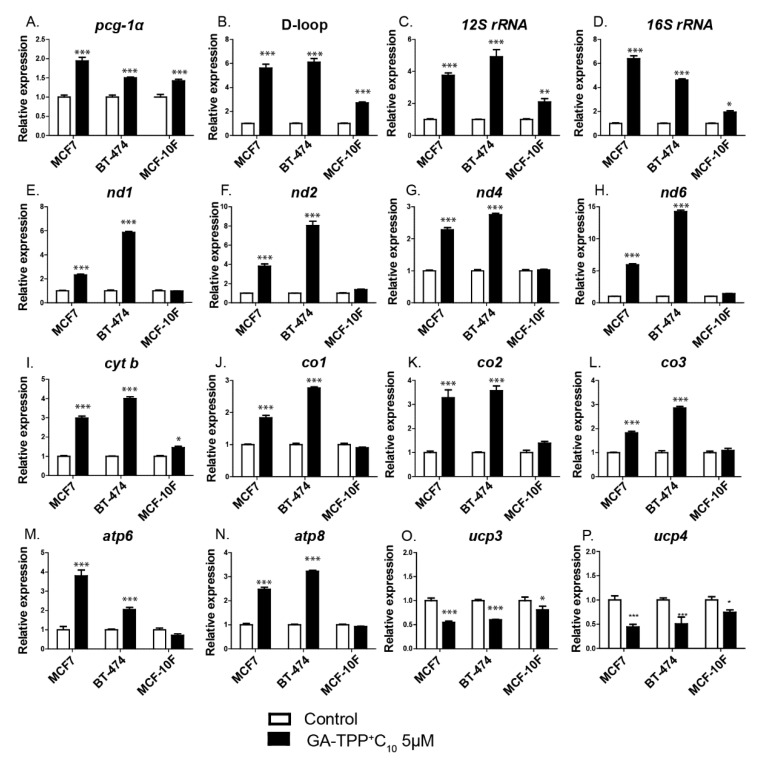
The effect of GA-TPP^+^C_10_ increases mitochondrial biogenesis-related gene expression in BC cells. (**A**–**N**) Changes in the expression of genes related to mitochondrial biogenesis and (**O**,**P**) uncoupling proteins (*ucp*s) induced by GA-TPP^+^C_10_ in the BC cells after 24 h of exposure. Values are expressed as the mean ± SEM of five independent experiments. * *p* < 0.05, ** *p* < 0.01, *** *p* < 0.001 vs. the control (DMSO).

We evaluated whether the metabolic stress induced by GA-TPP^+^C_10_ in cancer cells activates the expression of the transcription factor peroxisome proliferator-activated receptor gamma coactivator 1-alpha (*pgc-1α*) and some of its target genes as part of the adaptive response to GA-TPP^+^C_10_. As shown in Figure 6A–N, 5 μM GA-TPP^+^C_10_ increased the expression level of *pgc-1α*, mitochondrial DNA replication (shown by the amplification of the D-loop region) and all mitochondrial transcript levels (the ribosomal subunits *12S rRNA* and *16S rRNA*; the Complex I components *nd1*, *nd2*, *nd4*, and *nd6*; the Complex III components *cyt b*; the Complex IV components *mt-co1*, *mt-co2* and *mt-co3*, and the Complex V components *atp6* and *atp8*) in the BC cells at 24 h of exposure. Notably, although the MCF-10F epithelial cells showed a similar increase in the activity of *pgc-1α*, the number of mitochondria (D-loop) and the expression of ribosomal subunits after incubation with GA-TPP^+^C_10_, the levels of the subunits of the ETC complexes were not altered. Interestingly, GA-TPP^+^C_10_ induced a significant decrease in the levels of *ucp*s (*ucp3* and *ucp4*) in the MCF7, BT-474, and MCF-10F cells after 24 h (Figure 6O,P). All the results suggest a possible prosurvival adaptive mechanism based on the initiation of mitochondrial biogenesis and Δψ_m_ preservation by UCP downregulation under GA-TPP^+^C_10_-induced mitochondrial stress in cancer cells.

### 3.7. Doxy Selectively Inhibits the Synthesis of Mitochondrial Proteins Encoded by mt-DNA in BC Cells

We initially assessed the effect of Doxy on the translation of mRNAs in mitochondrial ribosomes (mt-ribosomes). To differentiate between the inhibition of the mt-ribosomes and the cytosolic ribosomes, we quantified the mRNA and protein levels of mitochondrial proteins encoded by both the nuclear genome (VDAC1 and UQCRC2) and the mitochondrial genome (mt-DN_1_ mt-CO_1_ and mt-CO_2_). As shown in Figure S7, after a 24 h incubation with increasing concentrations of Doxy, we observed a significant decrease in the levels of proteins synthesized in the mt-ribosomes. However, no change was observed in the mRNA and protein levels of the nuclear genome-encoded genes. Interestingly, analysis of the MCF-10F epithelial cell line showed no significant differences in the expression of any of the proteins analyzed. To verify that the decrease in the mitochondrial protein levels was due to an inhibitory action in the ribosomes and not due to a decrease in mitochondrial gene expression, we analyzed the mRNA levels of *nd_1_*, *mt-co_1_*, *mt-co_2_*, and *uqcrc2*. As shown in Figure S7E–I, no significant variations were observed in any of the transcripts. These results indicate that Doxy inhibits the synthesis of mitochondrial proteins encoded by mt-DNA in BC cells.

### 3.8. Inhibition of Mitochondrial Bioenergetics by GA-TPP^+^C_10_ and Mitochondrial Translation by Doxy Promotes Synergistic Cytotoxic Effects in BC Cells

**Figure 7:**
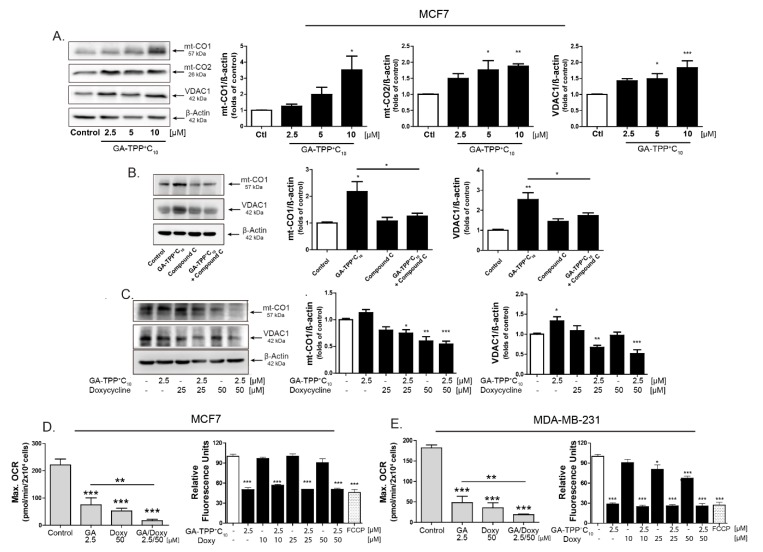
GA-TPP^+^C_10_ and Doxy combination decreases mitochondrial function in BC cells. (**A**) Effect of GA-TPP^+^C_10_, (**B**) the AMPK inhibitor Compound C and (C) the GA-TPP^+^C_10_ and Doxy combination on the mitochondrial protein levels of mt-CO_1_, mt-CO_2_, and VDAC after 24 h of exposure in MCF7 cells. (**C**,**D**) Effect of the combination on maximal OCR and ΔΨ_m_ after 24 h of exposure in MCF7 and MDA-MB-231 cells. * *p* < 0.05, ** *p* < 0.01, *** *p* < 0.001 vs. the control (DMSO).

We hypothesized that inhibition of the GA-TPP^+^C_10_-induced adaptive response involving increased expression of mitochondrial biogenesis-related genes may promote sensitization to cancer cell death. To evaluate this hypothesis, we combined GA-TPP^+^C_10_ with increasing concentrations of Doxy, and the changes in the mRNA and protein levels of mitochondrial components were evaluated after 24 h of exposure. Consistent with the above results, GA-TPP^+^C_10_ increased the expression of ETC-related genes (Figure 6), which translated into an increase in the expression of each mitochondrial protein encoded by the mt-DNA in the MCF7 (Figure 7A) and MDA-MB-231 cells (Figure S9A). Conversely, the MCF7 cells treated with Cpd. C and GA-TPP^+^C_10_ showed significantly decreased mitochondrial protein levels of mt-CO1 and VDAC, suggesting that the GA-TPP^+^C_10_-induced prosurvival response is mediated by AMPK-dependent mitochondrial biogenesis (Figure 7B). However, although the combined treatment with GA-TPP^+^C_10_ plus Doxy significantly increased the expression of ETC-related genes (Figure S8), the mt-CO1 and VDAC protein levels significantly decreased in both BC cell lines (Figure 7C and Figure S9B). In addition, the combination decreased the maximal mitochondrial respiration, promoting a reduction in the Δψm in BC cells (Figure 7D,E). These results suggest that the combination triggers decreases in mitochondrial function and mass.

**Figure 8:**
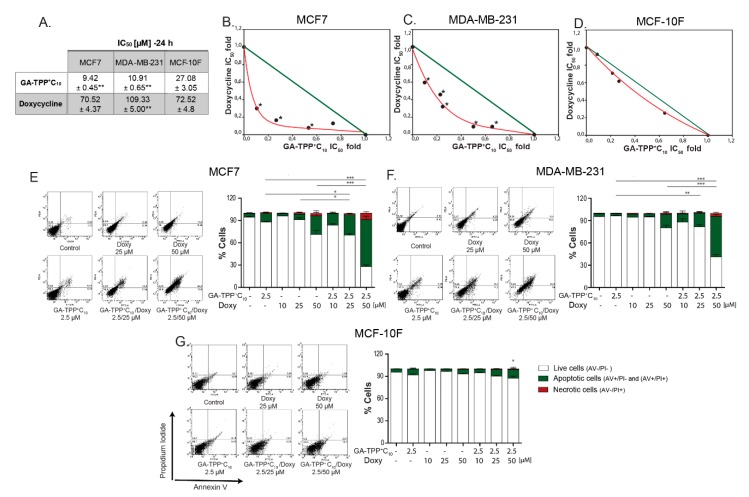
The GA-TPP^+^C_10_ and Doxy combination triggers a synergistic cytotoxic effect. (**A**–**D**) Analysis of the effect of the pharmacological interaction triggered by the GA-TPP^+^C_10_ plus Doxy combination on cell death in the BC and normal cells, (**E**–**G**) Quantification of the cell death induced by the GA-TPP^+^C_10_ and Doxy combination at 24 h of exposure. * *p* < 0.05, ** *p* < 0.01, *** *p* < 0.001 vs. the control (DMSO).

Given the IC_50_ values obtained at 24 h for GA-TPP^+^C_10_ and Doxy in the MCF7, MDA-MB-231, and MCF-10F cells (Figure 8A), low concentrations were chosen to evaluate the type of pharmacological interaction of the GA-TPP^+^C_10_ plus Doxy combination. According to the isobolograms, both MCF7 and MDA-MB-231 cells exhibited significant differences between the IC_50_ values obtained for each analyzed condition versus the theoretical additive effect. Similar values were obtained by MTT reduction and crystal violet assays (Figure S10). For nontumoral MCF-10F cells, these analyses showed no significant differences (Figure 8B–D). Moreover, 2.5 μM GA-TPP^+^C_10_ in combination with 10, 25, and 50 μM Doxy produced an AV-positive subpopulation accounting for 15.76%, 24.26%, and 65.54% of the MCF7 cells, respectively, and 11.78%, 20.90%, and 55.97% of the MDA-MB-231 cells, respectively. In all the conditions, the AV/PI+ subpopulation did not exceed 7.5%. For normal epithelial cells, this interaction was not observed, and the maximum percentage of cell death was 14.00% (Figure 8E–G). Therefore, our results indicated that the GA-TPP^+^C_10_ plus Doxy combination promotes selective and synergistic induction of cell death in BC cells.

## 4. Discussion

In BC, mitochondrial metabolism has been recognized as an essential factor that promotes metastasis [1,4,47], drug resistance [48,49,50], survival and propagation of cancer stem cells (CSCs) and tumor-initiating cells (TICs) [51,52], making it a promising target for new anticancer approaches.

Several studies have reported drug delivery systems for the transport of anticancer compounds to the mitochondria [18], highlighting the use of the lipophilic cations pyridinium [53,54,55] and TPP^+^ [56,57,58,59]. In particular, we showed that TPP^+^ derivatives of gallic acid and GA selectively induce cell death in BC cells [19,20] in a receptor status-independent manner [20] without toxic effects on nontumoral tissues in vivo [21]. Although the previously described effects are accompanied by alterations in mitochondrial bioenergetics, the mechanism of action of these TPP^+^-linked compounds on mitochondrial function remains unclear. In this work, we describe a complex mechanism of time-dependent mitochondrial dysfunction induced by GA-TPP^+^C_10_, which is involved in the cytotoxic effect, as previously reported by us [20]. Interestingly, our results suggest that in the initial phase (mitochondrial uptake), GA-TPP^+^C_10_ increases the state 4o respiration, leading to uncoupling of OXPHOS. During the following minutes of exposure, GA-TPP^+^C_10_ inhibits Complex I-dependent respiration, blocking the electron flux stimulated by FCCP, and after prolonged exposure (24 h), this compound produces notable inhibition of FCCP-stimulated respiration and αKGDHC activity. Previously, we described *para*- and *ortho*-hydroquinones [28,60,61] and alkyl gallate derivatives [62] as Complex I inhibitors that lacked this biphasic behavior. Conversely, TPP^+^-gallate derivatives exhibit an uncoupling effect of OXPHOS in isolated mitochondria and intact cancer cells, and this effect is sensitive to adenine nucleotide translocator (ANT) inhibition by atractyloside [19]; however, no instantaneous inhibitory effect of the ETC was observed. These results suggest that the direct interaction with ETC and, consequently, Complex I inhibition by GA-TPP^+^C_10_ is favored by TPP^+^.

αKGDHC is a TCA cycle enzyme composed of three subunits (E1, E2, and E3) that oxidizes and decarboxylates α-ketoglutarate and attaches coenzyme-A to the product to form succinyl-CoA [63]. Indeed, this enzyme is an essential gatekeeper of OXPHOS and in cancer cells modulates metabolic remodeling in response to tumoral bioenergetic requirements [64]. Although TPP^+^ derivatives are used to study mitochondrial function, high concentrations accumulating in the mitochondrial matrix lead to αKGDHC inhibition, which is characterized by a decrease in the Vmax and an increase in the Km [37]. This inhibition is enhanced by increasing the length of the alkyl side chain [37]. Our results showed that GA-TPP^+^C_10_ also produces mixed time-dependent inhibition of αKGDHC activity in the BC cells. Early observations regarding a direct interaction and functional dependence by measurement of the NAD^+^/NADH ratio between Complex I and αKGDHC [65,66], as well as the role of Complex I activity in the control of the proton-motive force for ATP-coupled respiration [42] and maintaining mitochondrial aspartate synthesis [40,41], suggest that the complex mitochondrial dysfunction induced by GA-TPP^+^C_10_ mediated by Complex I and αKGDHC inhibition blocks an essential step required for cancer cell survival and proliferation. Recent evidence indicates that selective Complex I inhibition decreases intracellular aspartate levels by decreasing the NAD^+^ pool, which is used as a substrate for αKGDHC for NADH regeneration in the TCA cycle, providing carbon units for DNA synthesis during proliferation [40,41]. Consequently, exogenous addition of Pyr to cancer cells treated with known ETC inhibitors rescues the aspartate levels and proliferation by generating NAD^+^ via oxidation to lactic acid. [67]. In this line, we showed that only supplementation with the cell-penetrating substrates dimethyl-α-ketoglutarate, an αKGDHC substrate, and methyl-aspartate, but not Pyr, partially rescued the cell death induced by GA-TPP^+^C_10_, suggesting that GA-TPP^+^C_10_ induces simultaneous inhibition of Complex I and αKGDHC.

The metabolic plasticity of cancer cells is known to favor acquired drug resistance and modulate prosurvival signaling pathways, allowing adaptation to changes in substrate availability [68,69,70]. GA-TPP^+^C_10_-induced mitochondrial dysfunction triggered early metabolic remodeling toward glycolysis, which may be mediated by AMPK signaling as previously described [29,71], and its inhibition with 2-DG promoted increased cell death in BC cells, similar to the reported synergistic effects of mitochondria-targeted antioxidants and vitamin E analogs and 2-DG in breast [72,73], hepatocellular [74] and pancreatic [75] carcinomas. Notably, we found that the induction of cell death with 2-DG plus GA-TPP^+^C_10_ treatment was less efficient than that of a combination with a panel of known chemotherapeutics, suggesting that mitochondrial inhibition enhances the efficacy of these agents.

The mitochondrial effect of GA-TPP^+^C_10_ also induced increased transcription of nuclear and mitochondrial genes related to the ETC components, mitochondrial ABC transporters and mitochondrial biogenesis, with decreased expression of UCP genes. Although GA-TPP^+^C_10_-induced cell death was less extensive in MCF7-Rho0 and MCF7-TAMR, two cell lines lacking ETC or decreased ETC activity, respectively [32], than in the parental MCF7 cell line, we observed an upregulation of the mitochondrial transporter gene *abcb7* in all cell lines. Recently, this transporter was shown to induce the hypoxia-independent accumulation of hypoxia-inducible factor 1 alpha (HIF-1α) and inhibit both apoptotic and nonapoptotic death in cancer cells [76]. Given that the energy sensor AMPK is involved in cellular metabolic control and regulates mitochondrial biogenesis via PGC1α phosphorylation under stress conditions in normal and malignant cells, we evaluated whether AMPK activation triggered by GA-TPP^+^C_10_-induced mitochondrial dysfunction may promote an adaptive response. The inhibition of GA-TPP^+^C_10_-dependent AMPK activation reduced the protein levels of mt-CO_1_ and VDAC and produced an increase in BC cell death. Therefore, our results suggest that the increases in the gene expression and protein levels of several mitochondrial components induced by GA-TPP^+^C_10_ are involved in a prosurvival phenotype mediated by AMPK signaling.

In this work, we hypothesized that to promote the extensive cell death induced by GA-TPP^+^C_10_ via blocking the mitochondrial dysfunction-induced adaptive response, the inhibition of mitochondrial protein translation by the Doxy combination may induce selective vulnerability in cancer cells. Doxy, a bacteriostatic antibiotic drug that inhibits the 30S subunit of the bacterial ribosome, can also exert inhibitory effects on the 28S subunit of the human mitochondrial ribosome due to the high homology of the two subunits [22,24,25]. The inhibition of mitochondrial protein synthesis triggered by Doxy has been documented in several organisms [77,78,79] and has been shown to promote the mitochondrial damage generated by an imbalance between the mitochondrial complex proteins encoded by the nuclear and mitochondrial genomes. This effect produces mitochondrial complex instability and decreased respiration. Consistent with this finding, we demonstrated that Doxy blocks the adaptive response induced by GA-TPP^+^C_10_, inhibiting the translation of ETC components and promoting selective and synergistic death in BC cells.

## 5. Conclusions

Our results describe the anticancer mechanism of GA-TPP^+^C_10_, a mitochondria-targeted hydroquinone that induces a complex inhibition of mitochondrial bioenergetics in a time-dependent manner in BC cells. Moreover, because evidence showed that the Doxy-induced mitonuclear protein imbalance does not generate manifest toxicity in animal models [79] and because we showed here that the combination of Doxy with GA-TPP^+^C_10_ produces synergistic lethality, we propose that this combined strategy based on the blockage of the mitochondrial bioenergetic inhibition-induced adaptive response may have therapeutic relevance in BC.

## Supplementary Materials

The following are available online at https://www.mdpi.com/2073-4409/9/2/407/s1, Figure S1: GA-TPP^+^C_10_ reduces the clonogenic potential of BC cells, Figure S2: Effect of GA-TPP^+^C_10_ and chemotherapeutics on the cell death of BC cells, Figure S3: Basal expression of PGC-1α and mitochondrial genes, Figure S4: GA-TPP^+^C_10_ triggers depolarization of the mitochondrial membrane potential, Figure S5: Inhibition of ETC induced by GA-TPP^+^C_10_, Figure S6: Glycolysis inhibition by 2-deoxy-D-glucose enhanced the cytotoxic effect induced by GA-TPP^+^C_10_ in BC cells, Figure S7: Doxycycline inhibits the translation of proteins encoded by only mt-DNA, Figure S8: The doxycycline + GA-TPP^+^C_10_ combination triggers an increase in ETC-related gene expression in BC cells, Figure S9: Doxycycline inhibits the compensatory response to the complex inhibition of mitochondrial bioenergetics induced by GA-TPP^+^C_10_ in MDA-MB-231 cells, Figure S10: The GA-TPP^+^C_10_ and Doxy combination triggers a synergistic cytotoxic effect. Table S1: Details of the primer sequences used in this study.

## Appendix A

**Detailed Protocols**

**MCF7-TAMR, MCF7-rho0 and MCF7-Sph cell cultures.**

Modified MCF7-TAMR cells were cultured in DMEM supplemented with 10% FBS, penicillin (100 IU/mL), streptomycin (100 μg/mL) and 5 μM tamoxifen. MCF7-Sph cells were obtained by maintaining parental MCF7 cells in advanced DMEM (Sigma-Aldrich) supplemented with NeuroCult Neural Stem Cell Proliferation Supplement (Stem Cell Technologies, Vancouver, British Columbia, Canada), rhEGF (20 ng/mL; Sigma-Aldrich), rhFGF (10 ng/mL; Sigma-Aldrich), penicillin (100 IU/mL) and streptomycin (100 μg/mL). Modified MCF7-rho0 cells were cultured in DMEM supplemented with 10% FBS, penicillin (100 IU/mL), streptomycin (100 μg/mL), uridine (50 μg/mL; Sigma-Aldrich) and sodium pyruvate (100 μg/mL; Sigma-Aldrich). All cell lines were maintained at 37 °C in a 5% CO_2_ atmosphere.

**RNA extraction, reverse transcription and qPCR**

Evaluation of the effect of GA-TPP^+^C_10_ (5 μM) on the levels of different mRNAs was performed by qPCR, as described by Truksa et al. [30]. Briefly, 1 × 10^6^ cells/mL were plated in a 100 mm Petri dish. Subsequently, the cells were treated with GA-TPP^+^C_10_ (5 μM) for 24 h. Total RNA was extracted by the RNAzol® T kit (Molecular Research Center, Inc., Cincinnati, OH, USA) according to the manufacturer’s instructions. Samples were stored at −80 °C until use. qPCR analysis was performed using the 5× HOT FIREpol Eva Green qPCR Mix Kit (Solis Biodyne, Tartu, Estonia) and an Illumina Eco Real-Time PCR System (Illumina, San Diego, CA, USA.). Briefly, 100 ng of cDNA was mixed with 5× Eva Green Mastermix (contained in the kit) and the appropriate primers (forward and reverse, 10 μM each). After the contents of the plate were mixed by inversion, the plate was incubated for 12 min at 95 °C for initial denaturation of the sample, and qPCR was performed according to the following protocol (40 cycles): 10 seconds at 95 °C (denaturation), 20 seconds at 60 °C (hybridization), and 20 seconds at 72 °C (extension and quantification). Quantification of the relative expression of each mRNA was performed by the ΔΔCt method, using the housekeeping genes (*polr2a* and *rplp0*) for comparison. The sequences of the primers used in this study are detailed in Table S1.

**Western blot analysis of AMPKα, phospho-AMPKα, mt-ND1, mt-CO1, mt-CO2, UQCRC2 and VDAC1**

For AMPKα and phospho-AMPKα detection, MCF7, MDA-MB-231 and MCF-10F cells were treated with GA-TPP^+^C_10_ (5–10 μM) for 4 and 24 h. For quantification of mitochondrial protein inhibition by doxycycline, MCF7, MDA-MB-231 and MCF-10F cells were treated with doxycycline (10–50 μM) for 24 h. Then, in both cases, the cells were washed in PBS and lysed with RIPA buffer (Tris−Cl [50 mM], NaCl [150 mM], sodium dodecyl sulfate [SDS; 0.1%]) containing proteinase and phosphatase inhibitor cocktail (Cell Signaling Technology, Danvers, MA, USA). The lysate was centrifuged at 20,000 g for 10 min at 4 °C. The protein concentrations were determined using a BCA Protein Assay Kit (Pierce, Rockford, IL, USA).

A 40-μg protein sample was separated by 12% SDS-polyacrylamide gel electrophoresis (SDS-PAGE), and the resolved proteins were transferred to a methylcellulose membrane (Millipore, Billerica, MA., USA. The membrane was blocked with 5% nonfat milk in Tris-buffered saline containing 0.1% Tween-20 (TBST) at room temperature for 1 h and then incubated with the primary antibodies at 4 °C overnight. After being washed with TBS-T, the membrane was incubated with anti-rabbit horseradish peroxidase-conjugated secondary antibodies. The following antibodies were used: antibodies against phospho-AMPKα (Thr172) (D79.5E, Cell Signaling, #4188), AMPKα (Cell Signaling, #2532), mt-DN1 (Abcam, ab74257), mt-CO1 (Abcam, ab14705), mt-CO2 (Abcam, ab110258), UQCRC2 (Abcam ab14745), VDAC1 (Abcam, ab15895) and β-actin (D6A8, Cell Signaling, #8457). All of these antibodies were used at a 1:1000 dilution with overnight incubation at 4 °C. The membranes were incubated with the secondary antibody (HRP) (goat anti-rabbit IgG-HRP, Santa Cruz Biotechnology, SC-2004, 1:5000) for 2 h. Finally, the membranes were exposed to the chemiluminescent reagent Luminata Forte Western HRP substrate (Millipore) and visualized using C-digit equipment (Li-Cor, Lincoln, NE, USA). The densitometric analysis was performed using ImageJ 1.47v software.

## References

[1] LeBleu V.S., O’Connell J.T., Gonzalez Herrera K.N., Wikman H., Pantel K., Haigis M.C., de Carvalho F.M., Damascena A., Domingos Chinen L.T., Rocha R.M. (2014). PGC-1alpha mediates mitochondrial biogenesis and oxidative phosphorylation in cancer cells to promote metastasis. Nat. Cell Biol..

[2] Bajzikova M., Kovarova J., Coelho A.R., Boukalova S., Oh S., Rohlenova K., Svec D., Hubackova S., Endaya B., Judasova K. (2018). Reactivation of Dihydroorotate Dehydrogenase-Driven Pyrimidine Biosynthesis Restores Tumor Growth of Respiration-Deficient Cancer Cells. Cell Metab..

[3] Maiuri M.C., Kroemer G. (2015). Essential role for oxidative phosphorylation in cancer progression. Cell Metab..

[4] Porporato P.E., Payen V.L., Perez-Escuredo J., De Saedeleer C.J., Danhier P., Copetti T., Dhup S., Tardy M., Vazeille T., Bouzin C. (2014). A mitochondrial switch promotes tumor metastasis. Cell Rep..

[5] Tan A.S., Baty J.W., Dong L.F., Bezawork-Geleta A., Endaya B., Goodwin J., Bajzikova M., Kovarova J., Peterka M., Yan B. (2015). Mitochondrial genome acquisition restores respiratory function and tumorigenic potential of cancer cells without mitochondrial DNA. Cell Metab..

[6] Dong L.F., Kovarova J., Bajzikova M., Bezawork-Geleta A., Svec D., Endaya B., Sachaphibulkij K., Coelho A.R., Sebkova N., Ruzickova A. (2017). Horizontal transfer of whole mitochondria restores tumorigenic potential in mitochondrial DNA-deficient cancer cells. eLife.

[7] Berridge M.V., McConnell M.J., Grasso C., Bajzikova M., Kovarova J., Neuzil J. (2016). Horizontal transfer of mitochondria between mammalian cells: Beyond co-culture approaches. Curr. Opin. Genet. Dev..

[8] Scarpulla R.C. (2011). Metabolic control of mitochondrial biogenesis through the PGC-1 family regulatory network. Biochim. ET Biophys. Acta.

[9] Birsoy K., Possemato R., Lorbeer F.K., Bayraktar E.C., Thiru P., Yucel B., Wang T., Chen W.W., Clish C.B., Sabatini D.M. (2014). Metabolic determinants of cancer cell sensitivity to glucose limitation and biguanides. Nature.

[10] Urra F.A., Weiss-López B., Araya-Maturana R. (2016). Determinants of Anti-Cancer Effect of Mitochondrial Electron Transport Chain Inhibitors: Bioenergetic Profile and Metabolic Flexibility of Cancer Cells. Curr. Pharm. Des..

[11] Lehuédé C., Dupuy F., Rabinovitch R., Jones R.G., Siegel P.M. (2016). Metabolic Plasticity as a Determinant of Tumor Growth and Metastasis. Cancer Res..

[12] van Weverwijk A., Koundouros N., Iravani M., Ashenden M., Gao Q., Poulogiannis G., Jungwirth U., Isacke C.M. (2019). Metabolic adaptability in metastatic breast cancer by AKR1B10-dependent balancing of glycolysis and fatty acid oxidation. Nat. Commun..

[13] Zaal E.A., Berkers C.R. (2018). The Influence of Metabolism on Drug Response in Cancer. Front. Oncol..

[14] Ghosh J.C., Siegelin M.D., Vaira V., Faversani A., Tavecchio M., Chae Y.C., Lisanti S., Rampini P., Giroda M., Caino M.C. (2015). Adaptive Mitochondrial Reprogramming and Resistance to PI3K Therapy. JNCI J. Natl. Cancer Inst..

[15] Fulda S., Galluzzi L., Kroemer G. (2010). Targeting mitochondria for cancer therapy. Nat. Rev. Drug Discov..

[16] Boukalova S., Rohlenova K., Rohlena J., Neuzil J., Oliveira P.J. (2018). Mitocans: Mitochondrially Targeted Anti-cancer Drugs. Mitochondrial Biology and Experimental Therapeutics.

[17] Modica-Napolitano J.S., Aprille J.R. (2001). Delocalized lipophilic cations selectively target the mitochondria of carcinoma cells. Adv. Drug Deliv. Rev..

[18] Battogtokh G., Cho Y.-Y., Lee J.Y., Lee H.S., Kang H.C. (2018). Mitochondrial-Targeting Anticancer Agent Conjugates and Nanocarrier Systems for Cancer Treatment. Front. Pharmacol..

[19] Jara J.A., Castro-Castillo V., Saavedra-Olavarria J., Peredo L., Pavanni M., Jana F., Letelier M.E., Parra E., Becker M.I., Morello A. (2014). Antiproliferative and uncoupling effects of delocalized, lipophilic, cationic gallic acid derivatives on cancer cell lines. Validation in vivo in singenic mice. J. Med. Chem..

[20] Sandoval-Acuna C., Fuentes-Retamal S., Guzman-Rivera D., Peredo-Silva L., Madrid-Rojas M., Rebolledo S., Castro-Castillo V., Pavani M., Catalan M., Maya J.D. (2016). Destabilization of mitochondrial functions as a target against breast cancer progression: Role of TPP(+)-linked-polyhydroxybenzoates. Toxicol. Appl. Pharmacol..

[21] Peredo-Silva L., Fuentes-Retamal S., Sandoval-Acuna C., Pavani M., Maya J.D., Castro-Castillo V., Madrid-Rojas M., Rebolledo S., Kemmerling U., Parra E. (2017). Derivatives of alkyl gallate triphenylphosphonium exhibit antitumor activity in a syngeneic murine model of mammary adenocarcinoma. Toxicol. Appl. Pharmacol..

[22] Chopra I., Roberts M. (2001). Tetracycline antibiotics: Mode of action, applications, molecular biology, and epidemiology of bacterial resistance. Microbiol. Mol. Biol. Rev. MMBR.

[23] Roberts M.C. (2003). Tetracycline therapy: Update. Clin. Infect. Dis..

[24] Lamb R., Ozsvari B., Lisanti C.L., Tanowitz H.B., Howell A., Martinez-Outschoorn U.E., Sotgia F., Lisanti M.P. (2015). Antibiotics that target mitochondria effectively eradicate cancer stem cells, across multiple tumor types: Treating cancer like an infectious disease. Oncotarget.

[25] Ozsvari B., Fiorillo M., Bonuccelli G., Cappello A.R., Frattaruolo L., Sotgia F., Trowbridge R., Foster R., Lisanti M.P. (2017). Mitoriboscins: Mitochondrial-based therapeutics targeting cancer stem cells (CSCs), bacteria and pathogenic yeast. Oncotarget.

[26] Tallarida R.J. (2001). Drug Synergism: Its Detection and Applications. J. Pharmacol. Exp. Ther..

[27] Franken N.A.P., Rodermond H.M., Stap J., Haveman J., van Bree C. (2006). Clonogenic assay of cells in vitro. Nat. Protoc..

[28] Urra F.A., Martinez-Cifuentes M., Pavani M., Lapier M., Jana-Prado F., Parra E., Maya J.D., Pessoa-Mahana H., Ferreira J., Araya-Maturana R. (2013). An ortho-carbonyl substituted hydroquinone derivative is an anticancer agent that acts by inhibiting mitochondrial bioenergetics and by inducing G(2)/M-phase arrest in mammary adenocarcinoma TA3. Toxicol. Appl. Pharmacol..

[29] Urra F.A., Munoz F., Cordova-Delgado M., Ramirez M.P., Pena-Ahumada B., Rios M., Cruz P., Ahumada-Castro U., Bustos G., Silva-Pavez E. (2018). FR58P1a; a new uncoupler of OXPHOS that inhibits migration in triple-negative breast cancer cells via Sirt1/AMPK/beta1-integrin pathway. Sci. Rep..

[30] Truksa J., Dong L.F., Rohlena J., Stursa J., Vondrusova M., Goodwin J., Nguyen M., Kluckova K., Rychtarcikova Z., Lettlova S. (2015). Mitochondrially targeted vitamin E succinate modulates expression of mitochondrial DNA transcripts and mitochondrial biogenesis. Antioxid. Redox Signal..

[31] Weigel M.T., Dahmke L., Schem C., Bauerschlag D.O., Weber K., Niehoff P., Bauer M., Strauss A., Jonat W., Maass N. (2010). In vitro effects of imatinib mesylate on radiosensitivity and chemosensitivity of breast cancer cells. BMC Cancer.

[32] Tomková V., Sandoval-Acuña C., Torrealba N., Truksa J. (2019). Mitochondrial fragmentation, elevated mitochondrial superoxide and respiratory supercomplexes disassembly is connected with the tamoxifen-resistant phenotype of breast cancer cells. Free Radic. Biol. Med..

[33] Porteous C.M., Menon D.K., Aigbirhio F.I., Smith R.A., Murphy M.P. (2013). P-glycoprotein (Mdr1a/1b) and breast cancer resistance protein (Bcrp) decrease the uptake of hydrophobic alkyl triphenylphosphonium cations by the brain. Biochim. ET Biophys. Acta.

[34] Robey R.W., Pluchino K.M., Hall M.D., Fojo A.T., Bates S.E., Gottesman M.M. (2018). Revisiting the role of ABC transporters in multidrug-resistant cancer. Nat. Rev. Cancer.

[35] Kumar V., Aneesh Kumar A., Sanawar R., Jaleel A., Santhosh Kumar T.R., Kartha C.C. (2019). Chronic Pressure Overload Results in Deficiency of Mitochondrial Membrane Transporter ABCB7 Which Contributes to Iron Overload, Mitochondrial Dysfunction, Metabolic Shift and Worsens Cardiac Function. Sci. Rep..

[36] Schaedler T.A., Faust B., Shintre C.A., Carpenter E.P., Srinivasan V., van Veen H.W., Balk J. (2015). Structures and functions of mitochondrial ABC transporters. Biochem. Soc. Trans..

[37] Elkalaf M., Tůma P., Weiszenstein M., Polák J., Trnka J. (2016). Mitochondrial Probe Methyltriphenylphosphonium (TPMP) Inhibits the Krebs Cycle Enzyme 2-Oxoglutarate Dehydrogenase. PLoS ONE.

[38] Trnka J., Elkalaf M., Anděl M. (2015). Lipophilic triphenylphosphonium cations inhibit mitochondrial electron transport chain and induce mitochondrial proton leak. PLoS ONE.

[39] De Francesco E.M., Ozsvari B., Sotgia F., Lisanti M.P. (2019). Dodecyl-TPP Targets Mitochondria and Potently Eradicates Cancer Stem Cells (CSCs): Synergy With FDA-Approved Drugs and Natural Compounds (Vitamin C and Berberine). Front. Oncol..

[40] Birsoy K., Wang T., Chen W.W., Freinkman E., Abu-Remaileh M., Sabatini D.M. (2015). An Essential Role of the Mitochondrial Electron Transport Chain in Cell Proliferation Is to Enable Aspartate Synthesis. Cell.

[41] Sullivan L.B., Gui D.Y., Hosios A.M., Bush L.N., Freinkman E., Vander Heiden M.G. (2015). Supporting Aspartate Biosynthesis Is an Essential Function of Respiration in Proliferating Cells. Cell.

[42] Urra F.A., Munoz F., Lovy A., Cardenas C. (2017). The Mitochondrial Complex(I)ty of Cancer. Front. Oncol..

[43] Patel D., Menon D., Bernfeld E., Mroz V., Kalan S., Loayza D., Foster D.A. (2016). Aspartate Rescues S-phase Arrest Caused by Suppression of Glutamine Utilization in KRas-driven Cancer Cells. J. Biol. Chem..

[44] Saqcena M., Mukhopadhyay S., Hosny C., Alhamed A., Chatterjee A., Foster D.A. (2015). Blocking anaplerotic entry of glutamine into the TCA cycle sensitizes K-Ras mutant cancer cells to cytotoxic drugs. Oncogene.

[45] Toyama E.Q., Herzig S., Courchet J., Lewis T.L., Losón O.C., Hellberg K., Young N.P., Chen H., Polleux F., Chan D.C. (2016). Metabolism. AMP-activated protein kinase mediates mitochondrial fission in response to energy stress. Science (N. Y.).

[46] Hardie D.G., Ross F.A., Hawley S.A. (2012). AMPK: A nutrient and energy sensor that maintains energy homeostasis. Nat. Rev. Mol. Cell Biol..

[47] Atlante S., Visintin A., Marini E., Savoia M., Dianzani C., Giorgis M., Sürün D., Maione F., Schnütgen F., Farsetti A. (2018). α-ketoglutarate dehydrogenase inhibition counteracts breast cancer-associated lung metastasis. Cell Death Dis..

[48] Fiorillo M., Sotgia F., Sisci D., Cappello A.R., Lisanti M.P. (2017). Mitochondrial “power” drives tamoxifen resistance: NQO1 and GCLC are new therapeutic targets in breast cancer. Oncotarget.

[49] Deblois G., Smith H.W., Tam I.S., Gravel S.-P., Caron M., Savage P., Labbé D.P., Bégin L.R., Tremblay M.L., Park M. (2016). ERRα mediates metabolic adaptations driving lapatinib resistance in breast cancer. Nat. Commun..

[50] Park S., Chang C.-Y., Safi R., Liu X., Baldi R., Jasper J.S., Anderson G.R., Liu T., Rathmell J.C., Dewhirst M.W. (2016). ERRα-Regulated Lactate Metabolism Contributes to Resistance to Targeted Therapies in Breast Cancer. Cell Rep..

[51] Fiorillo M., Sotgia F., Lisanti M.P. (2019). “Energetic” Cancer Stem Cells (e-CSCs): A New Hyper-Metabolic and Proliferative Tumor Cell Phenotype, Driven by Mitochondrial Energy. Front. Oncol..

[52] Zacksenhaus E., Shrestha M., Liu J.C., Vorobieva I., Chung P.E.D., Ju Y., Nir U., Jiang Z. (2017). Mitochondrial OXPHOS Induced by RB1 Deficiency in Breast Cancer: Implications for Anabolic Metabolism, Stemness, and Metastasis. Trends Cancer.

[53] Fantin V.R., Berardi M.J., Scorrano L., Korsmeyer S.J., Leder P. (2002). A novel mitochondriotoxic small molecule that selectively inhibits tumor cell growth. Cancer Cell.

[54] Reedy J.L., Hedlund D.K., Gabr M.T., Henning G.M., Pigge F.C., Schultz M.K. (2016). Synthesis and Evaluation of Tetraarylethylene-based Mono-, Bis-, and Tris(pyridinium) Derivatives for Image-Guided Mitochondria-Specific Targeting and Cytotoxicity of Metastatic Melanoma Cells. Bioconjug. Chem..

[55] Xu J., He H., Zhou L.-J., Liu Y.-Z., Li D.-W., Jiang F.-L., Liu Y. (2018). Pyridinium and indole orientation determines the mitochondrial uncoupling and anti-cancer efficiency of F16. Eur. J. Med. Chem..

[56] Murphy M.P. (2008). Targeting lipophilic cations to mitochondria. Biochim. ET Biophys. Acta (BBA) Bioenerg..

[57] Cheng G., Zielonka J., Ouari O., Lopez M., McAllister D., Boyle K., Barrios C.S., Weber J.J., Johnson B.D., Hardy M. (2016). Mitochondria-Targeted Analogues of Metformin Exhibit Enhanced Antiproliferative and Radiosensitizing Effects in Pancreatic Cancer Cells. Cancer Res..

[58] Cheng G., Zielonka J., Hardy M., Ouari O., Chitambar C.R., Dwinell M., Kalyanaraman B. (2019). Synergistic inhibition of tumor cell proliferation by metformin and mito-metformin in the presence of iron chelators. Oncotarget.

[59] Cheng G., Zhang Q., Pan J., Lee Y., Ouari O., Hardy M., Zielonka M., Myers C.R., Zielonka J., Weh K. (2019). Targeting lonidamine to mitochondria mitigates lung tumorigenesis and brain metastasis. Nat. Commun..

[60] Plaza C., Pavani M., Faundez M., Maya J., Morello A., Becker M., De Ioannes A., Cumsille M., Ferreira J. (2008). Inhibitory effect of nordihydroguaiaretic acid and its tetra-acetylated derivative on respiration and growth of adenocarcinoma TA3 and its multiresistant variant TA3MTX-R. Vivo.

[61] Jaña F., Faini F., Lapier M., Pavani M., Kemmerling U., Morello A., Maya J.D., Jara J., Parra E., Ferreira J. (2013). Tumor cell death induced by the inhibition of mitochondrial electron transport: The effect of 3-hydroxybakuchiol. Toxicol. Appl. Pharmacol..

[62] Frey C., Pavani M., Cordano G., Muñoz S., Rivera E., Medina J., Morello A., Diego Maya J., Ferreira J. (2007). Comparative cytotoxicity of alkyl gallates on mouse tumor cell lines and isolated rat hepatocytes. Comp. Biochem. Physiol. Part A Mol. Integr. Physiol..

[63] Qi F., Pradhan R.K., Dash R.K., Beard D.A. (2011). Detailed kinetics and regulation of mammalian 2-oxoglutarate dehydrogenase. BMC Biochem..

[64] Vatrinet R., Leone G., De Luise M., Girolimetti G., Vidone M., Gasparre G., Porcelli A.M. (2017). The α-ketoglutarate dehydrogenase complex in cancer metabolic plasticity. Cancer Metab..

[65] Porpaczy Z., Sumegi B., Alkonyi I. (1987). Interaction between NAD-dependent isocitrate dehydrogenase, alpha-ketoglutarate dehydrogenase complex, and NADH:ubiquinone oxidoreductase. J. Biol. Chem..

[66] Sumegi B., Srere P.A. (1984). Complex I binds several mitochondrial NAD-coupled dehydrogenases. J. Biol. Chem..

[67] Van Vranken J.G., Rutter J. (2015). You Down With ETC? Yeah, You Know D!. Cell.

[68] Avagliano A., Ruocco M.R., Aliotta F., Belviso I., Accurso A., Masone S., Montagnani S., Arcucci A. (2019). Mitochondrial Flexibility of Breast Cancers: A Growth Advantage and a Therapeutic Opportunity. Cells.

[69] Cannino G., Ciscato F., Masgras I., Sánchez-Martín C., Rasola A. (2018). Metabolic Plasticity of Tumor Cell Mitochondria. Front. Oncol..

[70] Jia D., Lu M., Jung K.H., Park J.H., Yu L., Onuchic J.N., Kaipparettu B.A., Levine H. (2019). Elucidating cancer metabolic plasticity by coupling gene regulation with metabolic pathways. Proc. Natl. Acad. Sci. USA.

[71] Liemburg-Apers D.C., Wagenaars J.A.L., Smeitink J.A.M., Willems P.H.G.M., Koopman W.J.H. (2016). Acute stimulation of glucose influx upon mitoenergetic dysfunction requires LKB1, AMPK, Sirt2 and mTOR–RAPTOR. J. Cell Sci..

[72] Cheng G., Zielonka J., Dranka B.P., McAllister D., Mackinnon A.C., Joseph J., Kalyanaraman B. (2012). Mitochondria-targeted drugs synergize with 2-deoxyglucose to trigger breast cancer cell death. Cancer Res..

[73] Cheng G., Zielonka J., McAllister D.M., Mackinnon A.C., Joseph J., Dwinell M.B., Kalyanaraman B. (2013). Mitochondria-targeted vitamin E analogs inhibit breast cancer cell energy metabolism and promote cell death. BMC Cancer.

[74] Dilip A., Cheng G., Joseph J., Kunnimalaiyaan S., Kalyanaraman B., Kunnimalaiyaan M., Gamblin T.C. (2013). Mitochondria-targeted antioxidant and glycolysis inhibition: Synergistic therapy in hepatocellular carcinoma. Anticancer Drugs.

[75] Cheng G., Zielonka J., McAllister D., Hardy M., Ouari O., Joseph J., Dwinell M.B., Kalyanaraman B. (2015). Antiproliferative effects of mitochondria-targeted cationic antioxidants and analogs: Role of mitochondrial bioenergetics and energy-sensing mechanism. Cancer Lett..

[76] Kim J.Y., Kim J.-K., Kim H. (2019). ABCB7 simultaneously regulates apoptotic and non-apoptotic cell death by modulating mitochondrial ROS and HIF1α-driven NFκB signaling. Oncogene.

[77] Ahler E., Sullivan W.J., Cass A., Braas D., York A.G., Bensinger S.J., Graeber T.G., Christofk H.R. (2013). Doxycycline alters metabolism and proliferation of human cell lines. PLoS ONE.

[78] Houtkooper R.H., Mouchiroud L., Ryu D., Moullan N., Katsyuba E., Knott G., Williams R.W., Auwerx J. (2013). Mitonuclear protein imbalance as a conserved longevity mechanism. Nature.

[79] Moullan N., Mouchiroud L., Wang X., Ryu D., Williams E.G., Mottis A., Jovaisaite V., Frochaux M.V., Quiros P.M., Deplancke B. (2015). Tetracyclines Disturb Mitochondrial Function across Eukaryotic Models: A Call for Caution in Biomedical Research. Cell Rep..

