# Supplementary material for: Complex Mitochondrial Dysfunction Induced by TPP+-Gentisic Acid and Mitochondrial Translation Inhibition by Doxycycline Evokes Synergistic Lethality in Breast Cancer Cells
Source: Cells. 2020 Feb 11;9(2):407. doi: 10.3390/cells9020407 (PMC7072465; doi:10.3390/cells9020407)
Supplement: Supplementary file 1 [file cells-09-00407-s001.zip › Supplementary_Material.docx]

Supplementary Materials:

Complex Mitochondrial Dysfunction Induced by TPP^+^-Gentisic Acid and Mitochondrial Translation Inhibition by Doxycycline Evokes Synergistic Lethality in Breast Cancer Cells

Sebastián Fuentes-Retamal, Cristian Sandoval-Acuña, Liliana Peredo-Silva, Daniela Guzmán-Rivera, Mario Pavani, Natalia Torrealba, Jaroslav Truksa, Vicente Castro-Castillo, Mabel Catalán, Ulrike Kemmerling, Félix A. Urra, Jorge Ferreira.


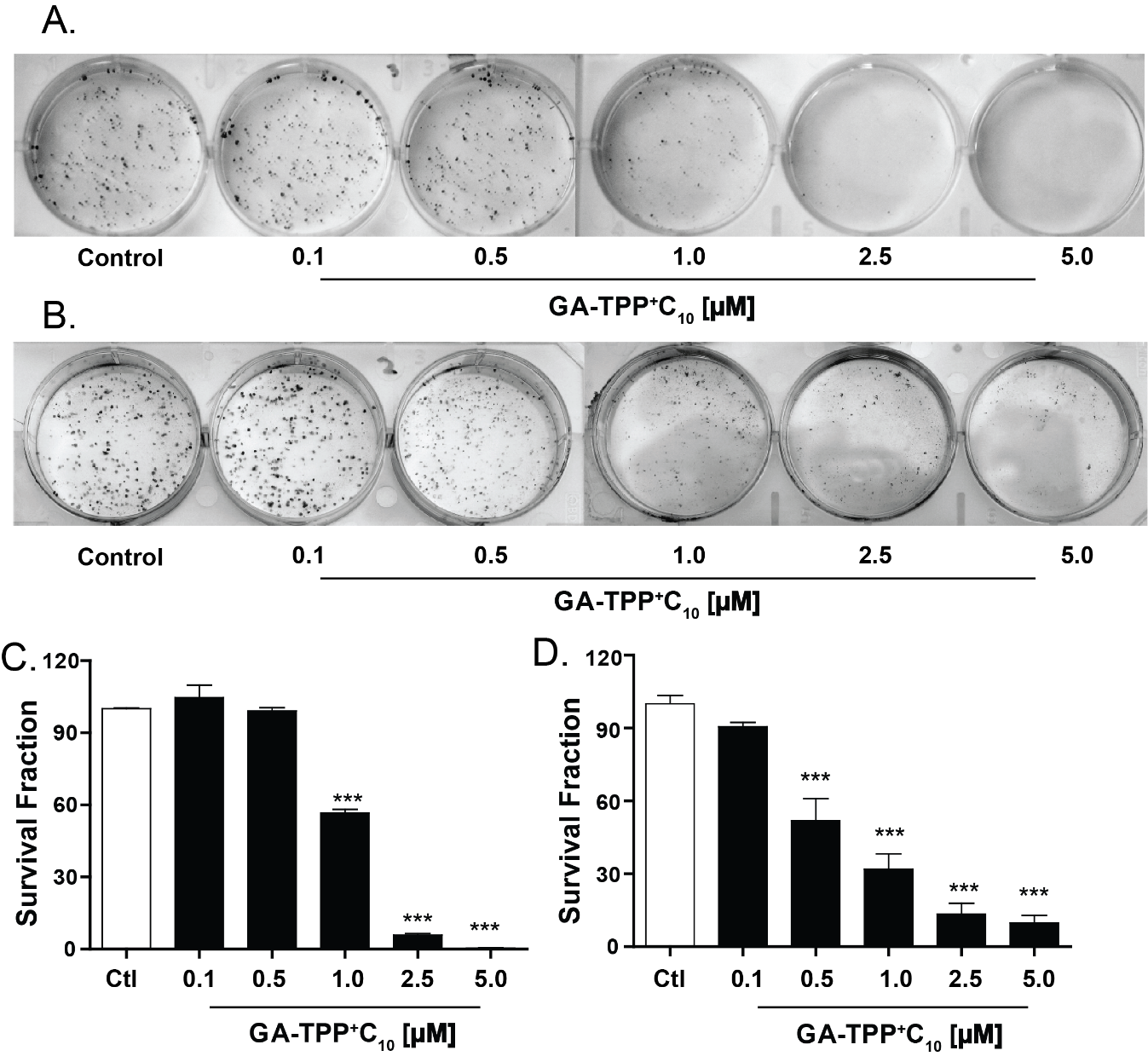


**Figure S1. GA-TPP^+^C_10_ reduces the clonogenic potential of BC cells.** (A-C) Representative images and quantification of colonies for the MCF7 and (B-D) MDA-MB-231 cells. Data are expressed as the mean ± SEM of three independent experiments. p<0.001 vs the control (DMSO).


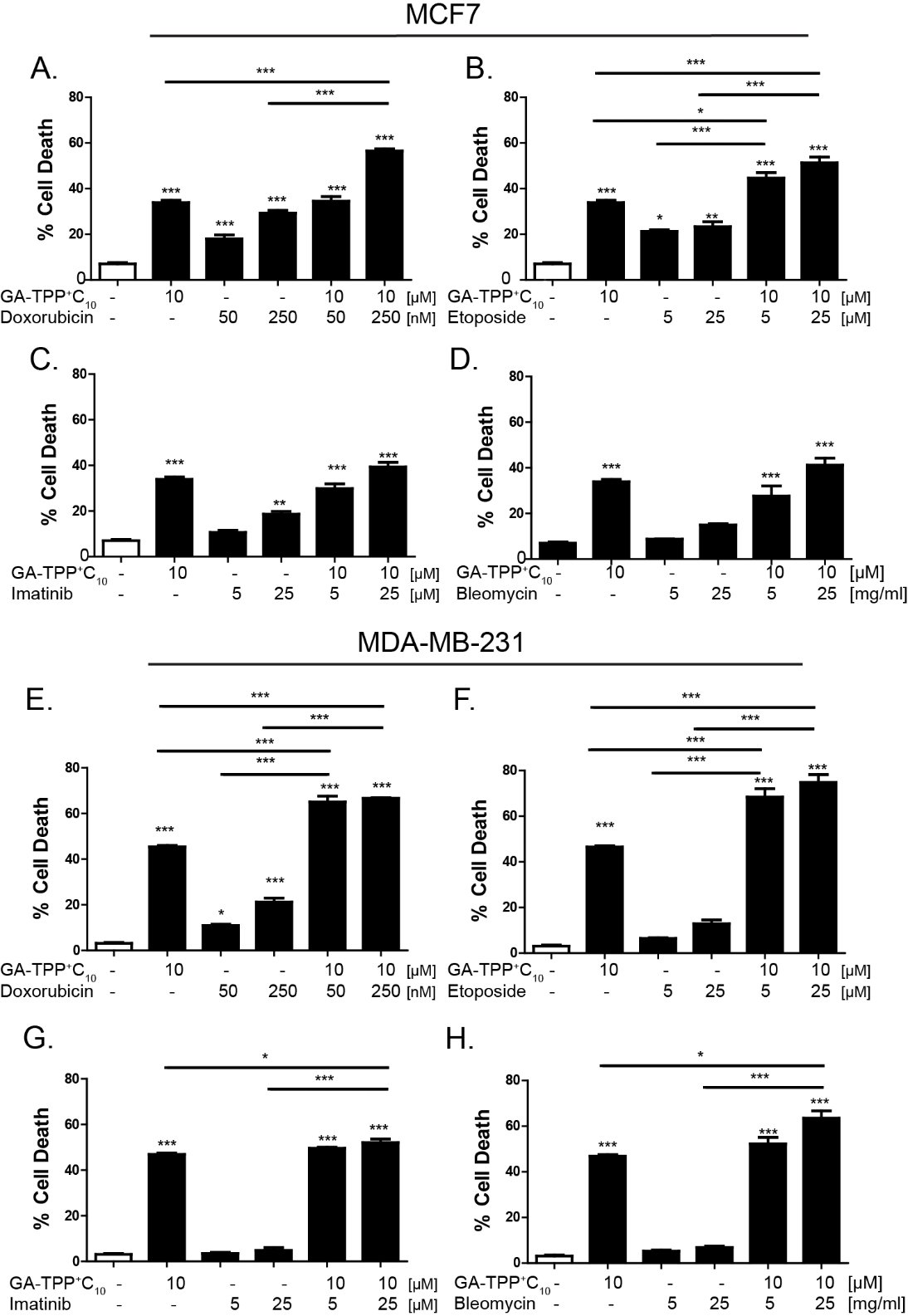


**Figure S2. Effect of GA-TPP^+^C_10_ and chemotherapeutics on the cell death of BC cells.** (A-D) Quantification of the cell death induced by the combination of GA-TPP^+^C_10_ and doxorubicin, etoposide, imatinib or bleomycin in MCF7 and (E-H) MDA-MB-231 cells at 24 h of exposure. Cell death was measured by flow cytometry as described in the Materials and Methods section. Data are expressed as the mean ± SEM of three independent experiments. *p<0.05, **p<0.01, ***p<0.001 vs the control (DMSO).


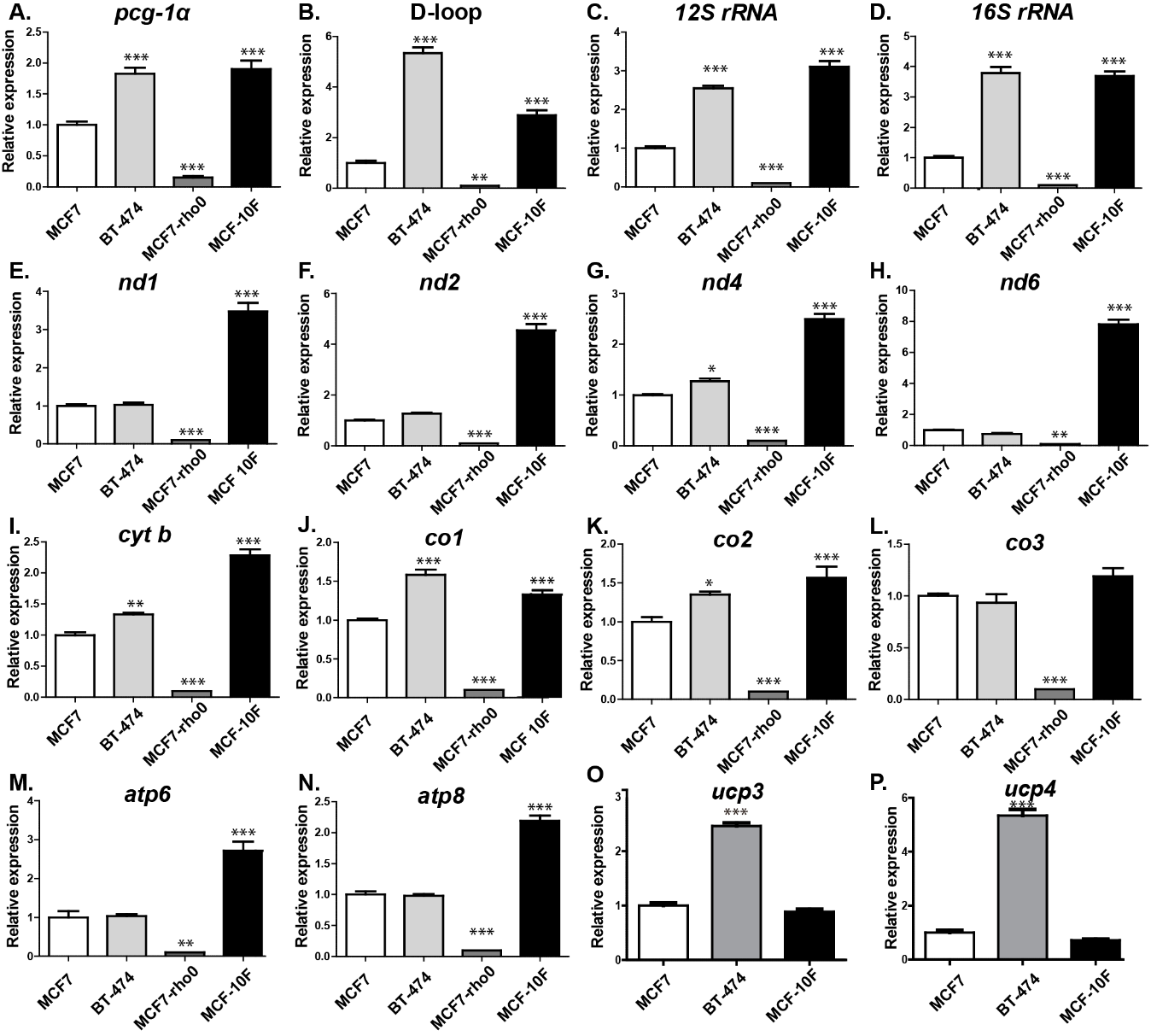


**Figure S3. Basal expression of PGC-1α and mitochondrial genes.** (A-N) Quantification of the basal expression of *pgc-1α*, D-loop and transcripts encoded by mt-DNA in MCF7, BT-474, MCF7-rho0 and MCF-10F cells. (O-P) Basal transcripts of *ucp* proteins in MCF7, BT-474, and MCF-10F cells. p<0.05. Data are expressed as the mean ± SEM of three independent experiments. **p<0.01, ***p<0.001 vs MCF7.


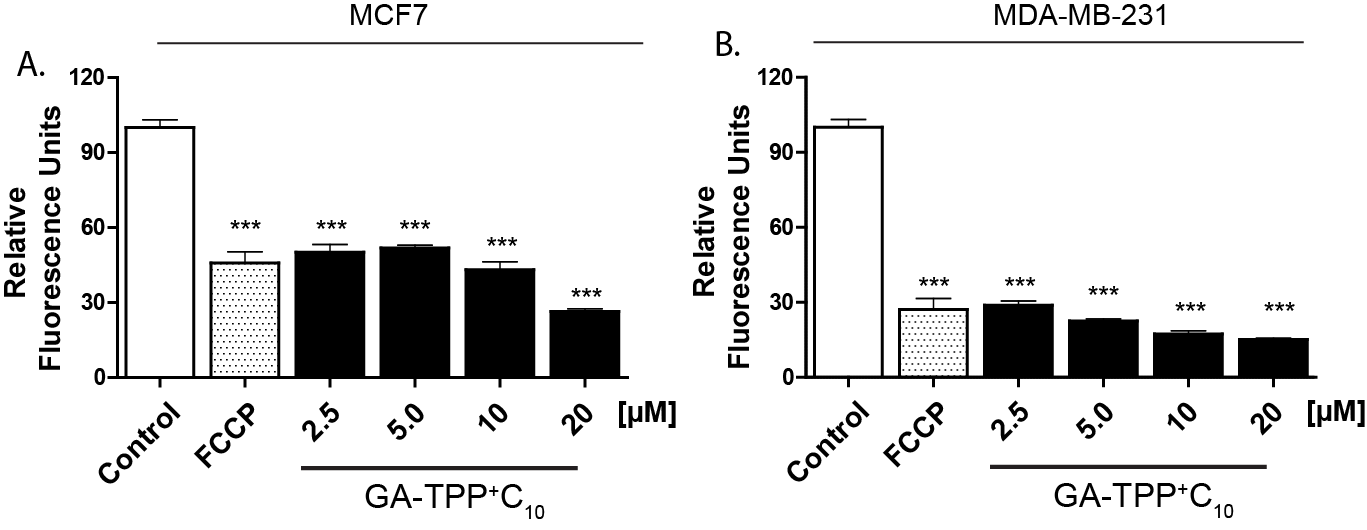


**Figure S4. GA-TPP^+^C_10_ triggers depolarization of the mitochondrial membrane potential.** (A) Effect induced by GA-TPP^+^C_10_ and FCCP (1 μM) on ΔΨ_m_ after 24 h of exposure in MCF7 and (B) MDA-MB-231 cells. Data are expressed as the mean ± SEM of three independent experiments. *p<0.05, **p<0.01, ***p<0.001 vs the control (DMSO).


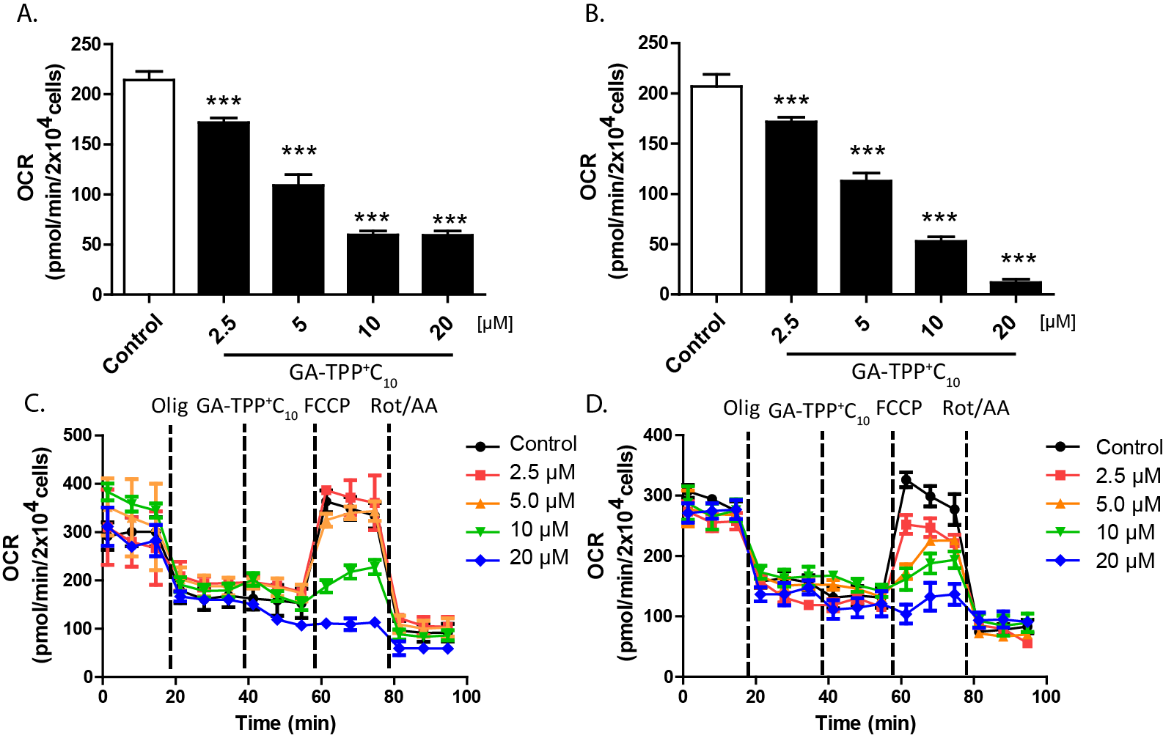


**Figure S5. Inhibition of ETC induced by GA-TPP^+^C_10_.** (A-B) Effect induced by GA-TPP^+^C_10_ on the basal OCR in MCF7 and MDA-MB-231 cells. (C-D) Effect induced by GA-TPP^+^C_10_ on the OCR in the presence of oligomycin in MCF7 and MDA-MB-231 cells. Data are expressed as the mean ± SEM of three independent experiments. ***p<0.001 vs the control (DMSO).


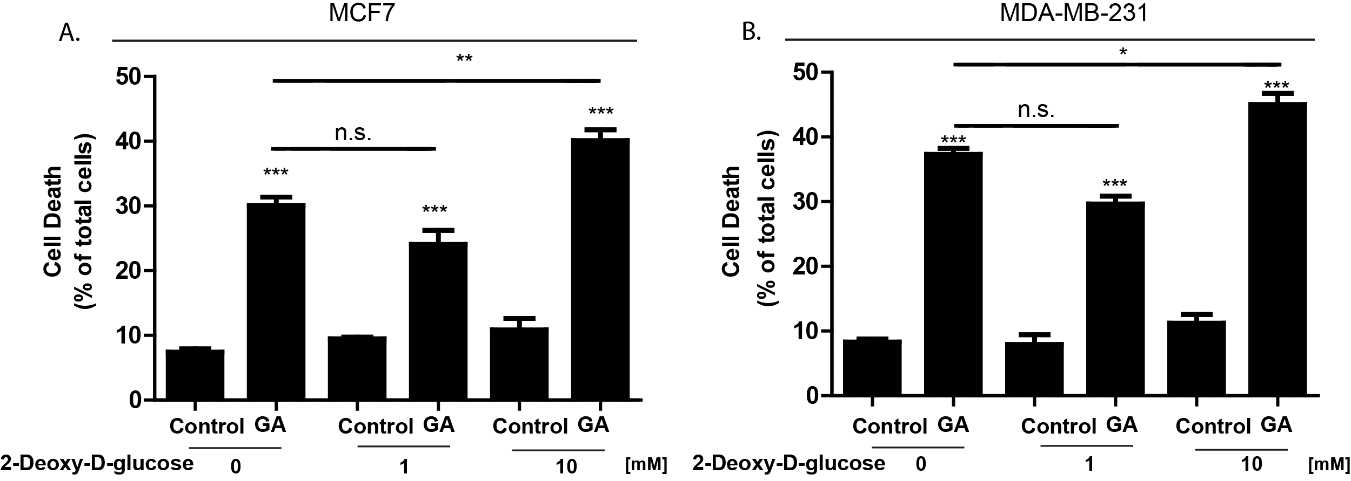


**Figure S6. Glycolysis inhibition by 2-deoxy-D-glucose enhanced the cytotoxic effect induced by GA-TPP^+^C_10_ in BC cells_._** (A) Effect of the combination of 10 μM GA-TPP^+^C_10_ and 2-deoxyglucose on the induction of cell death in MCF7 and (B) MDA-MB231 cells upon exposure for 24 h. Data are expressed as the mean ± SEM of three independent experiments. *p<0.05, **p<0.01, ***p<0.001 vs the control (DMSO).


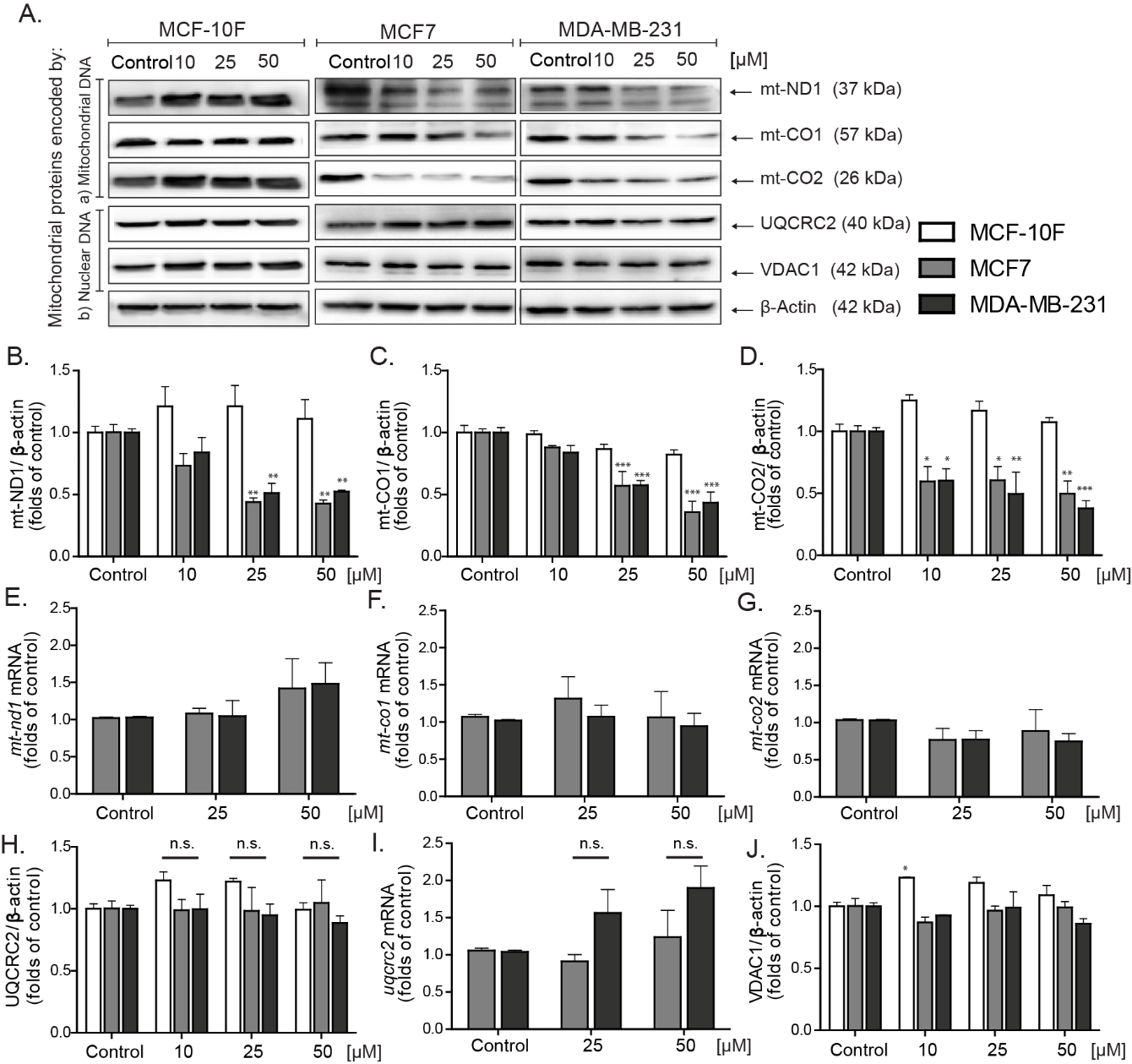


**Figure S7.** **Doxycycline inhibits the translation of proteins encoded by only mt-DNA:** (A-J) Changes induced by doxycycline in the levels of mRNAs and proteins encoded by nuclear DNA and mt-DNA after 24 h of exposure in BC cells and epithelial cells. Values are expressed as the mean ± SEM of five independent experiments. *p<0.05, **p<0.01, ***p<0.001 vs the control (DMSO).


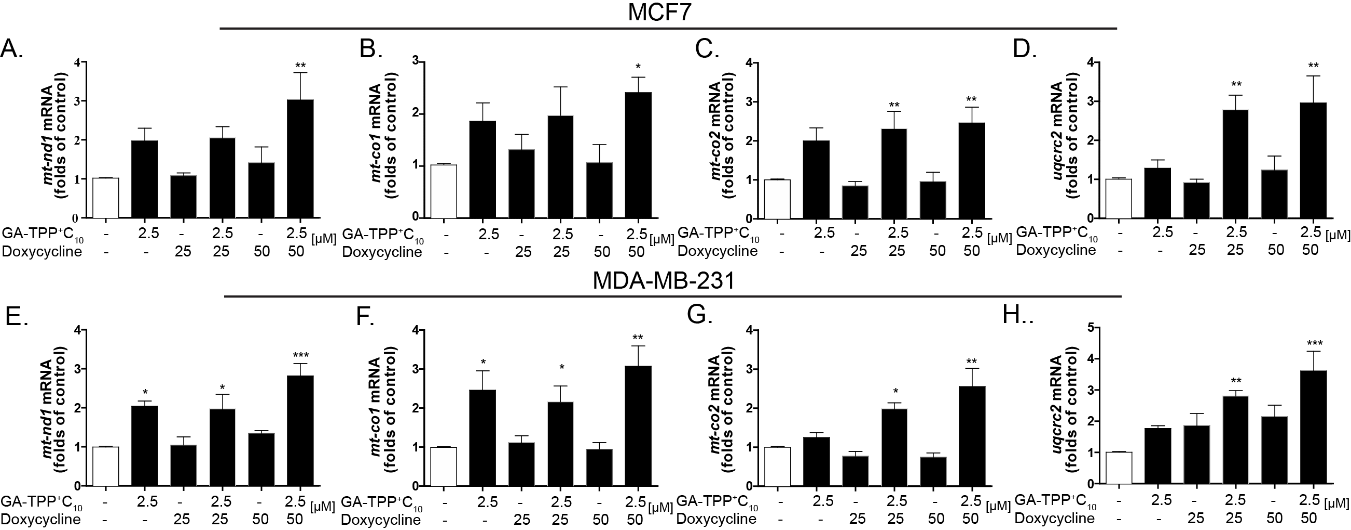


**Figure S8. The doxycycline + GA-TPP^+^C_10_ combination triggers an increase in ETC-related gene expression in BC cells**. (A-D) Changes in the mRNA levels of ETC components induced by the combination after 24 h of exposure in MCF7 and (E-H) MDA-MB-231 cells. Data are expressed as the mean ± SEM of three independent experiments. *p<0.05, **p<0.01, ***p<0.001 vs the control (DMSO).


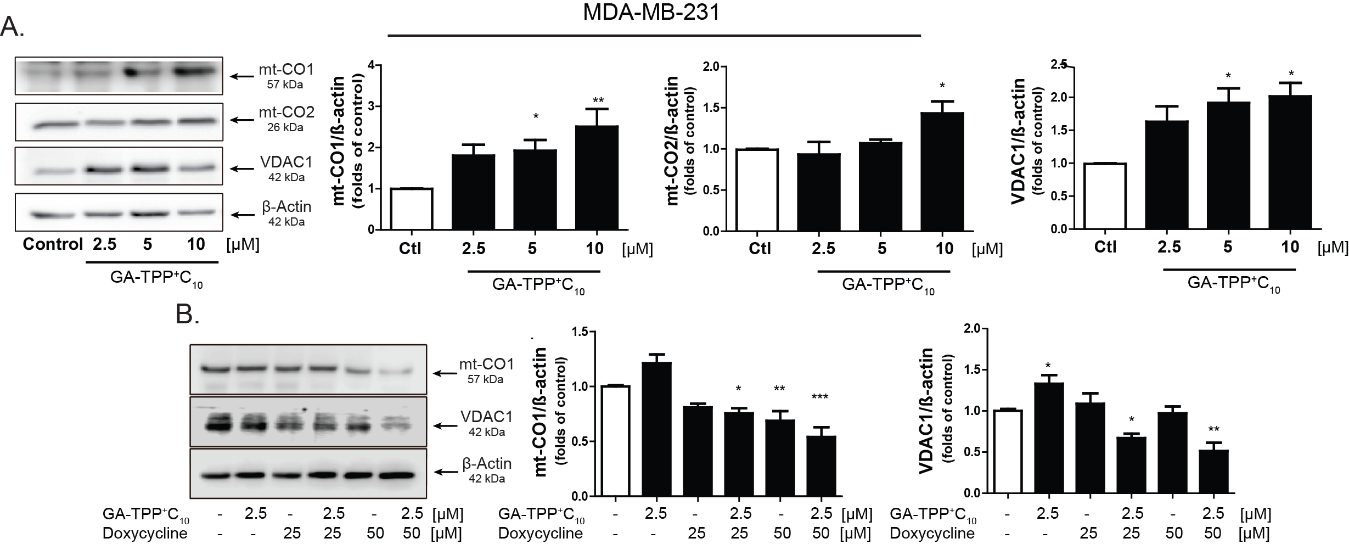


**Figure S9. Doxycycline inhibits the compensatory response to the complex inhibition of mitochondrial bioenergetics induced by GA-TPP^+^C_10_ in MDA-MB-231 cells.** (A) Effect of GA-TPP^+^C_10_ and (B) the combination of GA-TPP^+^C_10_ with doxycycline mitochondrial protein levels of mt-CO1, mt-CO2 and VDAC after 24 h of exposure in MDA-MB-231 cells. Data are expressed as the mean ± SEM of three independent experiments. *p<0.05, **p<0.01, ***p<0.001 vs the control (DMSO).


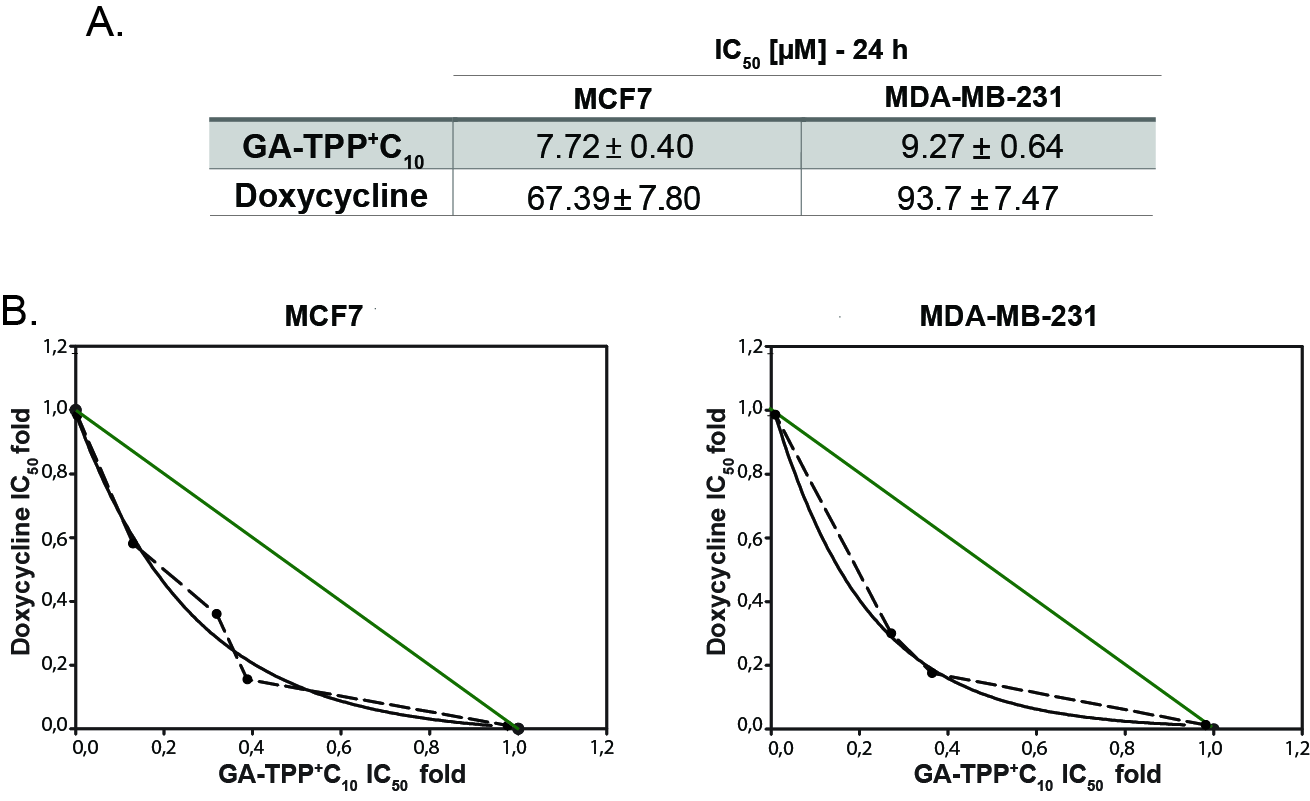


**Figure S10. The GA-TPP^+^C_10_ and Doxy combination triggers a synergistic cytotoxic effect.** (A-B) Analysis of the effect of the pharmacological interaction triggered by the GA-TPP^+^C_10_ plus Doxy combination on cell death in BC cells. Data were obtained from crystal violet assays and are expressed as the mean ± SEM of three independent experiments.

**Table S1.** Details of the primer sequences used in this study

| Gene | Forward | Reverse |
| --- | --- | --- |
| *ampk* | GTA CCA GGT CAT CAG TAC ACC A | CCT TCG TGG AGC CTG TTT TT |
| *ucp3* | GAA GGT CCG ATT TCA GGC CA | GTA GGT CAC CAC CTC AGC AC |
| *ucp4* | CGC TAC TGC TGA ATG TCC GT | GTC TCC CAA CCG AGC AAG AG |
| *ppargc1a* | GGG TGG ACT CAA GTG GTG CAG TG | GGT GGA GGG GTG CCG TCA GG |
| D-loop | TCG CCT GTA ATA TTG AAC GT | ATT TGG TAT TTT CGT CTG GG |
| *12s* | ACC ACC TCT TGC TCA GCC TA | CAT GGG CTA CAC CTT GAC CT |
| *16s* | TCC CGA TGG TGC AGC CGC TA | ACG GGG GAA GGC GCT TTG TG |
| *nd1* | ATA CCC ATG GCC AAC CTC CT | GGG CCT TTG CGT AGT TGT AT |
| *nd2* | GGC CCA ACC CGT CAT CTA CT | GAT GCG GTT GCT TGC GTG AG |
| *nd4* | ACT ACT CAC TCT CAC TGC CC | AGT GGA GTC CGT AAA GAG GT |
| *nd6* | CCT ACC TCC ATC GCT AAC CC | AGG GGG AAT GAT GGT TGT CT |
| *cyt b* | GAA ACT TCG GCT CAC TCC TT | GGC GAT TGA TGA AAA GGC GG |
| *co1* | GCC TCC GTA GAC CTA ACC AT | GTT ATG GCA GGG GGT TTT AT |
| *co2* | AGT CCT GTA TGC CCT TTT CC | GCG ATG AGG ACT AGG ATG AT |
| *co3* | CCC ACC AAT CAC ATG CCT AT | TAG GCC GGA GGT CAT TAG GA |
| *atp6* | CTG TTC GCT TCA TTC ATT GC | GAT TAG TCA TTG TTG GGT GG |
| *atp8* | TGC CCC AAC TAA ATA CTA CC | CTT TGG TGA GGG AGG TAG GT |
| *uqcrc2* | AAA GTT GCC CCG AAG GTT AAA | GAG CAT AGT TTT CCA GAG AAG CA |
| *porlr2a* | TGC TCC GTA TTC GCA TCA TGA ACA | ATC TGT CAG CAT GTT GGAA CTC GAT G |
| *rplp0* | ATC ACA GAG GAA ACT CTG CAT TCT CG | GAT AGA ATG GGG TAC TGA TGC AAC AGT T |
