# Supplementary figures and images for: Complex Mitochondrial Dysfunction Induced by TPP+-Gentisic Acid and Mitochondrial Translation Inhibition by Doxycycline Evokes Synergistic Lethality in Breast Cancer Cells
Source: Cells. 2020 Feb 11;9(2):407. doi: 10.3390/cells9020407 (PMC7072465; doi:10.3390/cells9020407)

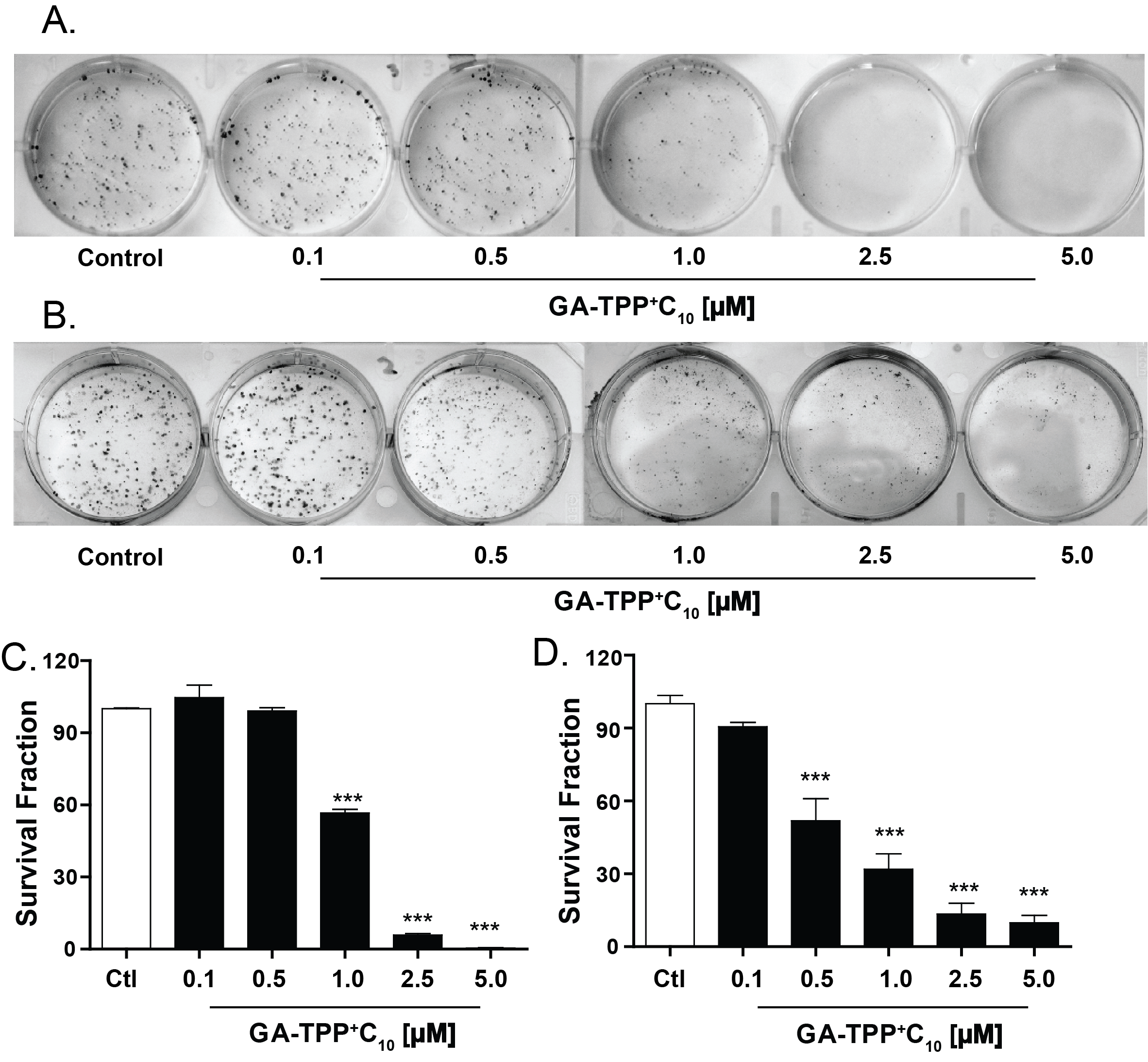

Supplement: Supplementary file 1 [file cells-09-00407-s001.zip › Figure S1.tif]

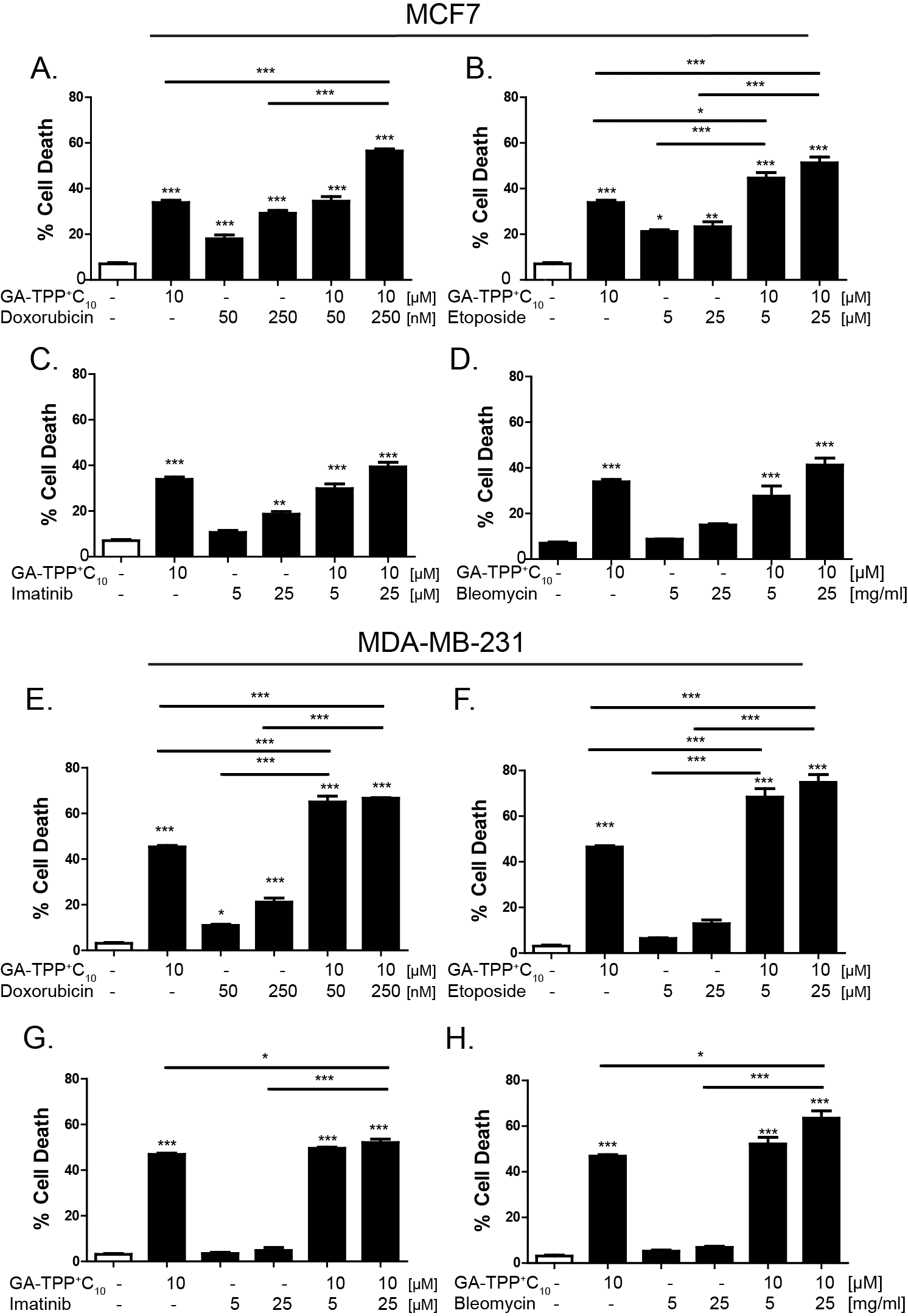

Supplement: Supplementary file 1 [file cells-09-00407-s001.zip › Figure S2.tif]

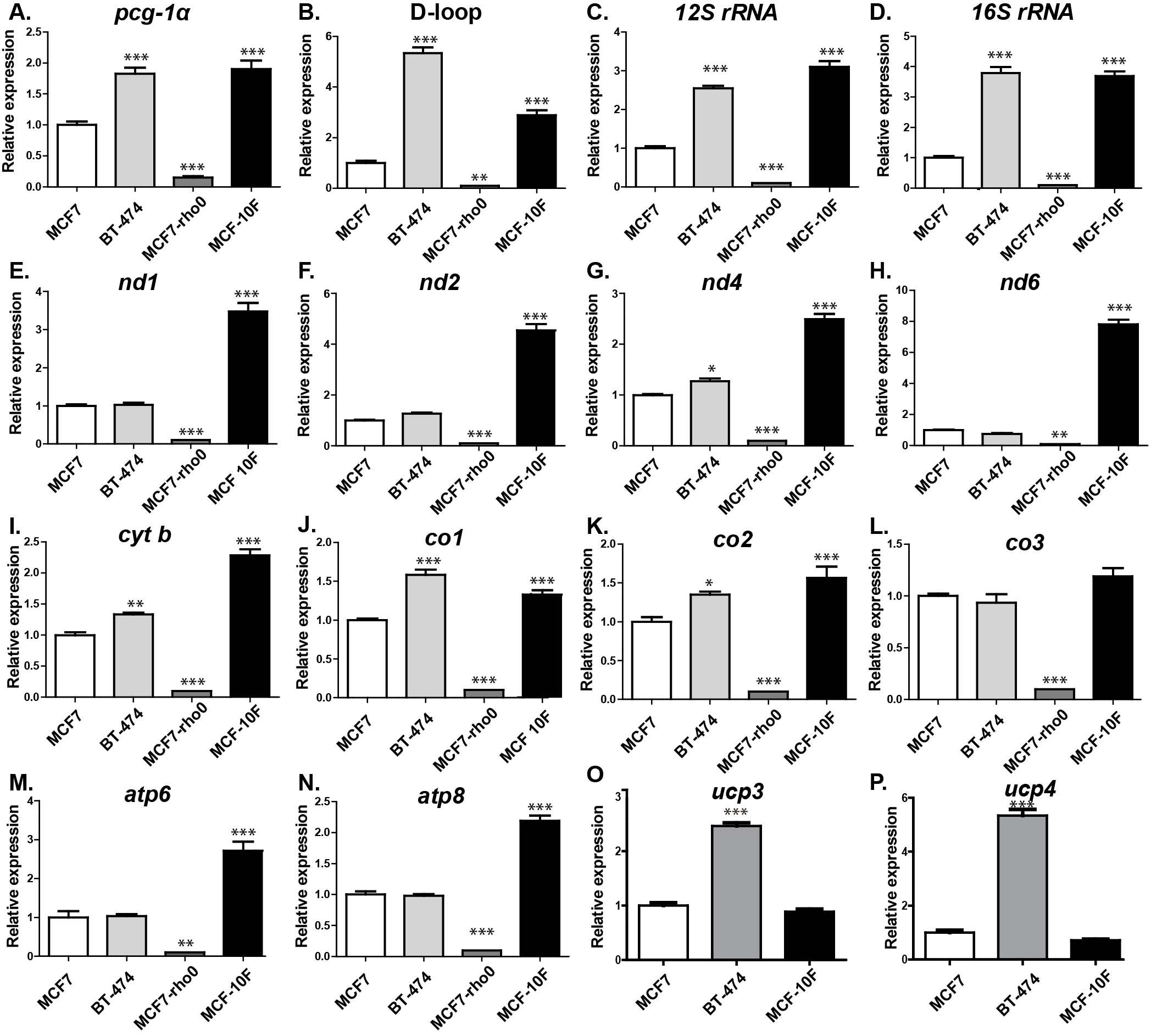

Supplement: Supplementary file 1 [file cells-09-00407-s001.zip › Figure S3.tif]

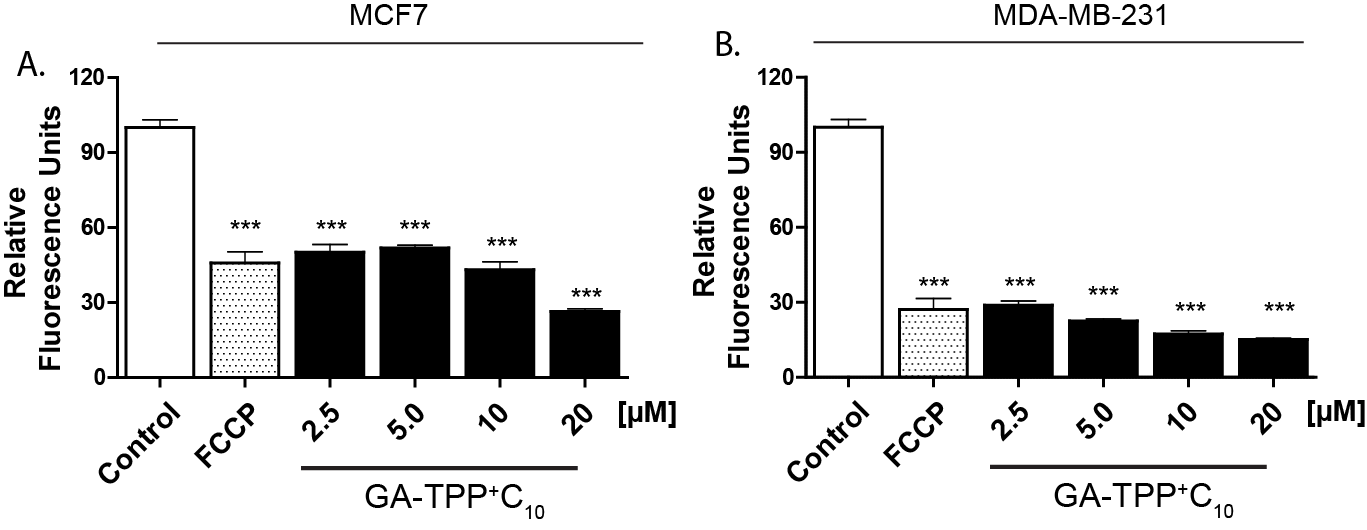

Supplement: Supplementary file 1 [file cells-09-00407-s001.zip › Figure S4.tif]

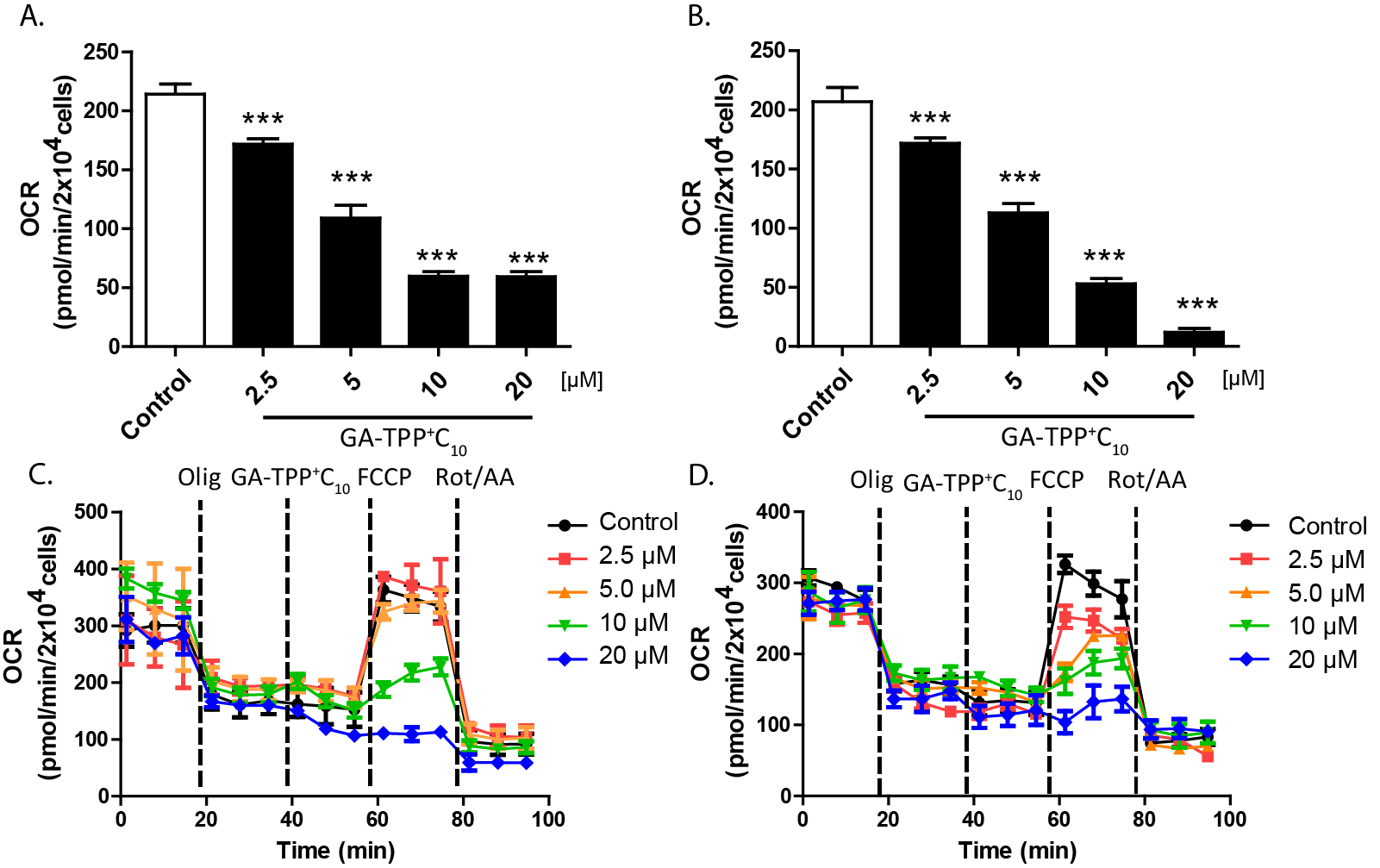

Supplement: Supplementary file 1 [file cells-09-00407-s001.zip › Figure S5.tif]

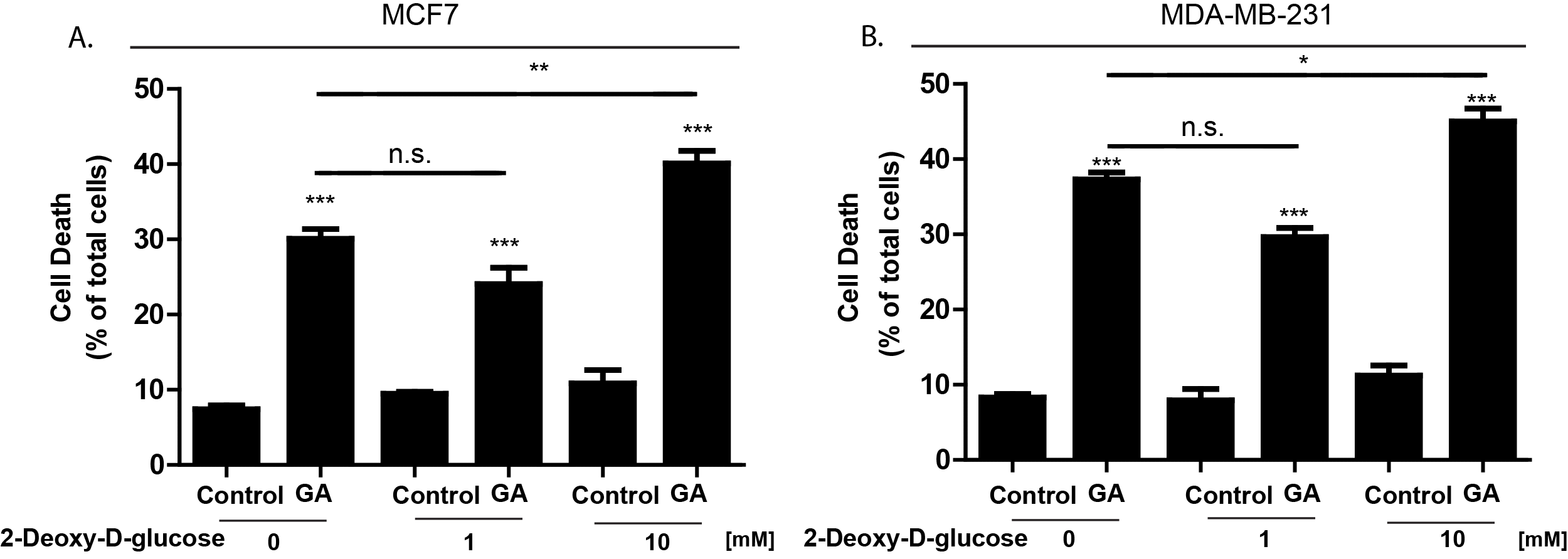

Supplement: Supplementary file 1 [file cells-09-00407-s001.zip › Figure S6.tif]

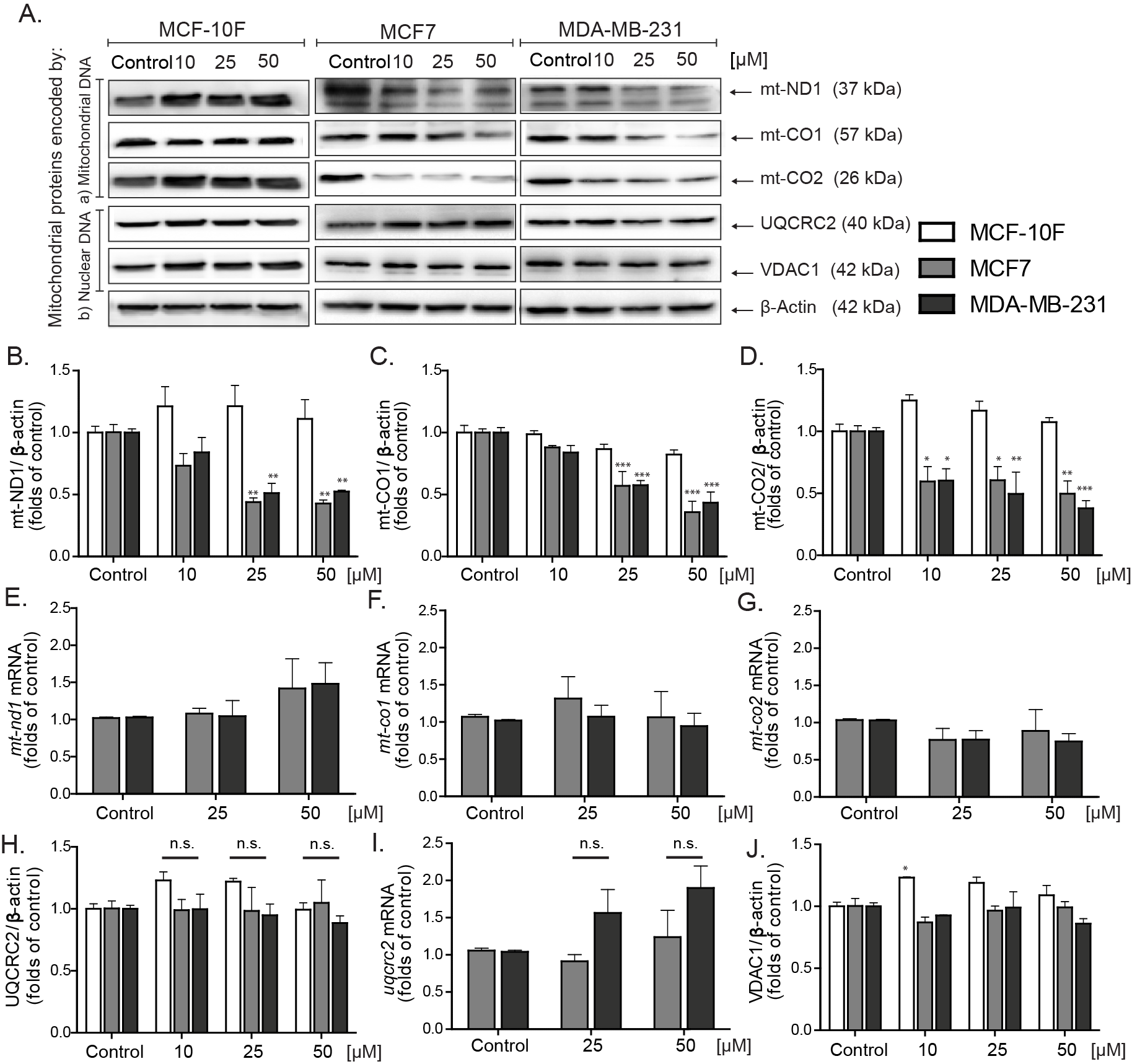

Supplement: Supplementary file 1 [file cells-09-00407-s001.zip › Figure S7.tif]

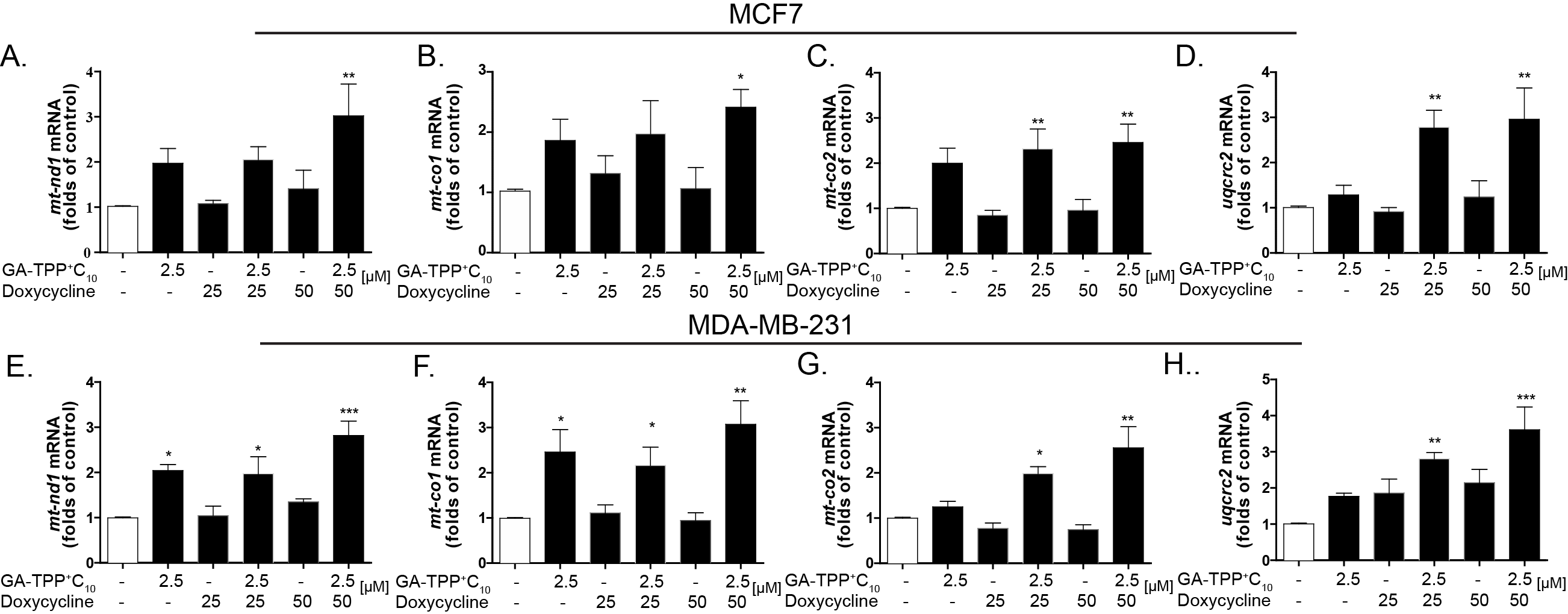

Supplement: Supplementary file 1 [file cells-09-00407-s001.zip › Figure S8.tif]

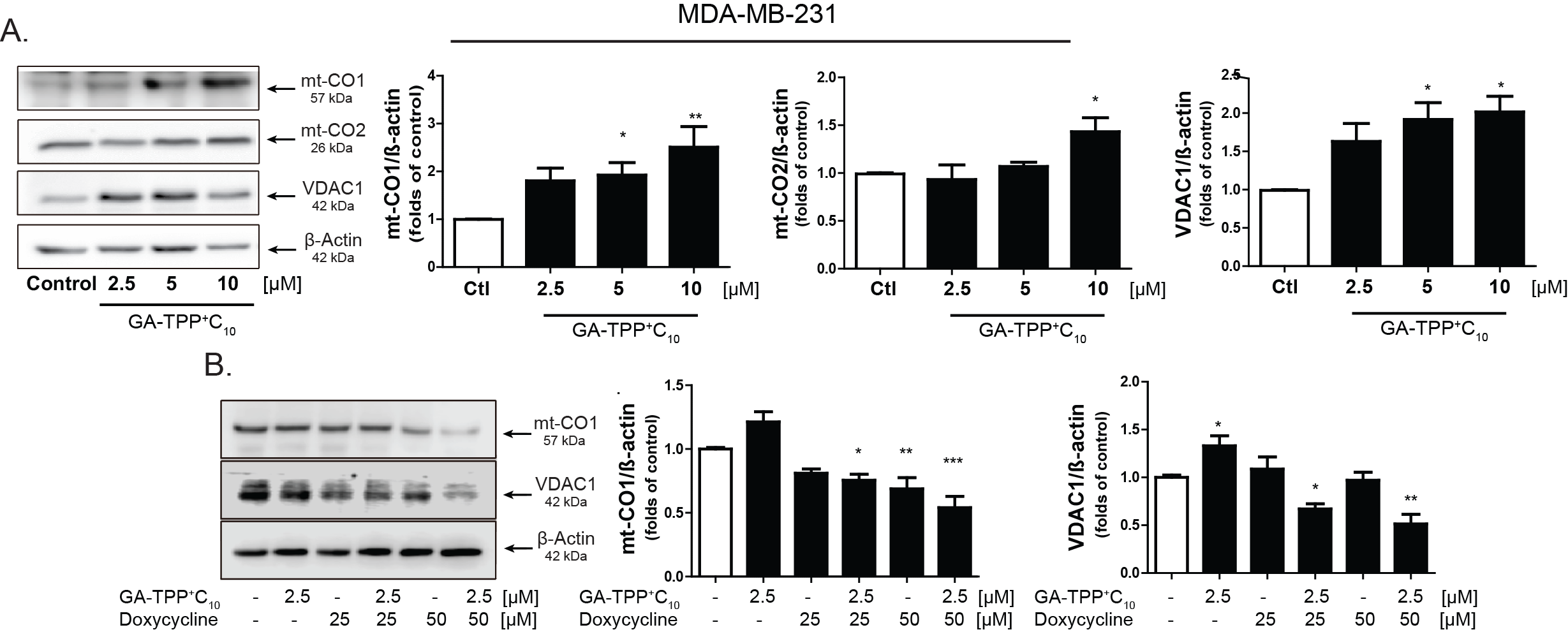

Supplement: Supplementary file 1 [file cells-09-00407-s001.zip › Figure S9.tif]

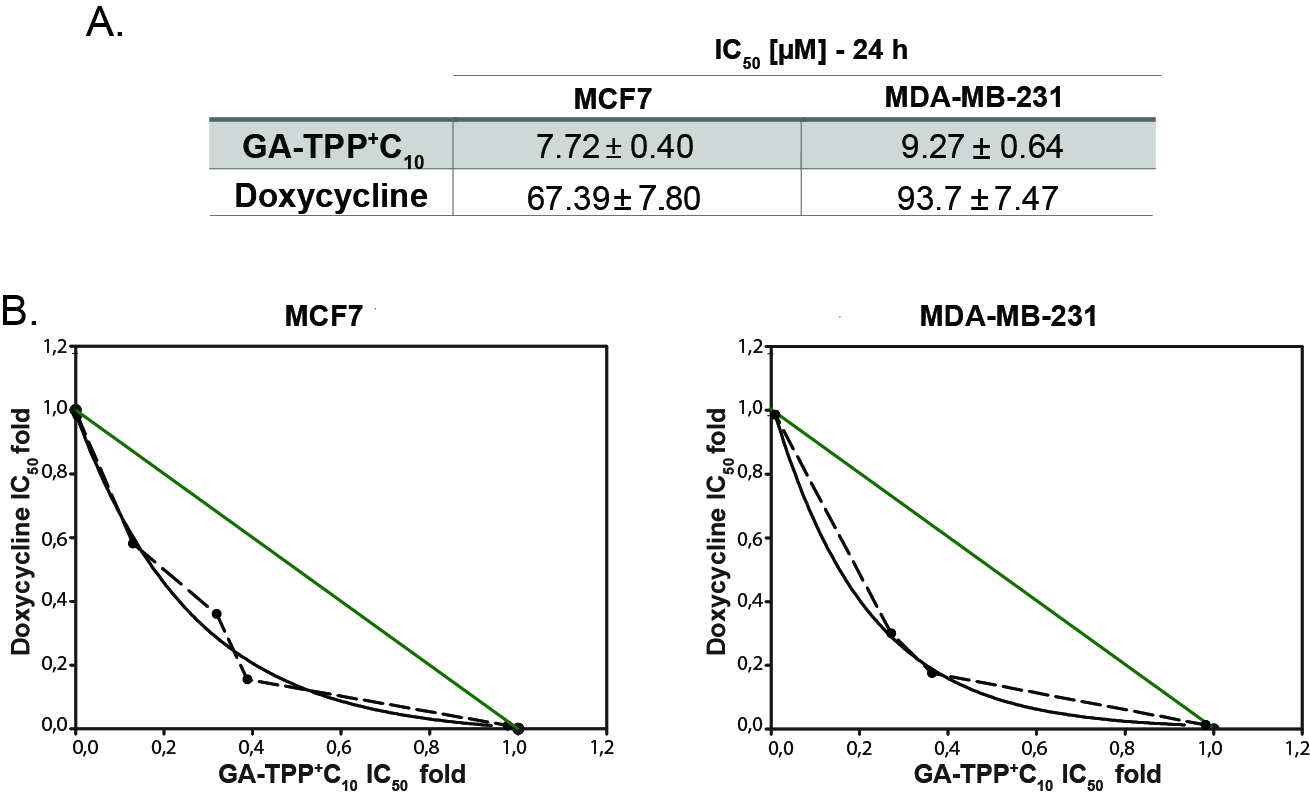

Supplement: Supplementary file 1 [file cells-09-00407-s001.zip › Figure S10.tif]
